# Remodeling of the focal adhesion complex by hydrogen-peroxide-induced senescence

**DOI:** 10.1038/s41598-023-36347-2

**Published:** 2023-06-15

**Authors:** Carolin Grandy, Fabian Port, Meytal Radzinski, Karmveer Singh, Dorothee Erz, Jonas Pfeil, Dana Reichmann, Kay-Eberhard Gottschalk

**Affiliations:** 1 Institute of Experimental Physics, Ulm University, 89081 Ulm, Baden-Württemberg Germany; 2 Department of Dermatology and Allergic Diseases, Ulm University, 89081 Ulm,, Baden-Württemberg Germany; 3 Alexander Silberman Institute of Life Science, The Hebrew University of Jerusalem, Edmond J. Safra Campus-Givat Ram, 9190401 Jerusalem, Israel

**Keywords:** Biological physics, Nanoscale biophysics

## Abstract

Cellular senescence is a phenotype characterized by cessation of cell division, which can be caused by exhaustive replication or environmental stress. It is involved in age-related pathophysiological conditions and affects both the cellular cytoskeleton and the prime cellular mechanosensors, focal adhesion complexes. While the size of focal adhesions increases during senescence, it is unknown if and how this is accompanied by a remodeling of the internal focal adhesion structure. Our study uses metal-induced energy transfer to study the axial dimension of focal adhesion proteins from oxidative-stress-induced senescent cells with nanometer precision, and compares these to unstressed cells. We influenced cytoskeletal tension and the functioning of mechanosensitive ion channels using drugs and studied the combined effect of senescence and drug intervention on the focal adhesion structure. We found that H_2_O_2_-induced restructuring of the focal adhesion complex indicates a loss of tension and altered talin complexation. Mass spectroscopy-based proteomics confirmed the differential regulation of several cytoskeletal proteins induced by H_2_O_2_ treatment.

## Introduction

The interaction of cells with the extracellular matrix regulates their morphology, growth, migration, and differentiation^1–3^. Focal adhesions (FA) are protein complexes that specialize in maintaining cell-ECM interactions, which enable cells to adhere to and interact with their environment. They are involved in cell-ECM adhesion, cytoskeletal regulation, signaling, and force transmission^4,5^ and consist of a layered, highly complex network of a multitude of proteins^6,7^. The actin cytoskeleton is a central element in focal adhesion since motor proteins such as non-muscle myosin II act on it, generating tension. It is connected to integrins via adaptor proteins such as talin or vinculin, which transmit the forces generated by motor proteins^6,8,9^. Paxillin is an important component at the interface between the integrin signaling and force transduction layers^6^. Talin binds integrins and actin. Force leads to the partial unfolding of talin, exposing hidden vinculin-binding sites^10^. Vinculin therefore plays a major role in the force transduction layer and is considered a force sensor^8,10^. The membrane is the baseline of the focal adhesion and integrins are anchored to it^6^. The actin regulatory layer, located above the force transduction layer, contains actin as well as actin-crosslinking proteins, such as alpha-actinin^6,7^. Tension is required for maturation of focal adhesions^11^. In the absence of tension, these complexes remain in a provisional state, so-called focal complexes, which are much smaller than focal adhesions and lack a large number of signaling proteins^12^. Focal adhesion maturation under force and ROCK activity in the Rho-ROCK pathway are closely connected^9,13^. Focal adhesion regulates the balance between cellular tension via motor proteins and adhesion tension through the binding of integrins to the extracellular matrix^4,14,15^. Previously, we showed that modulation of the ROCK pathway has a significant effect on focal adhesion structure^16^. Since the Rho-associated protein kinases ROCK 1 and ROCK 2 play key roles in cellular processes affected by aging^17^, we hypothesized that senescence alters the focal adhesion structure. In this study, we measured the vertical location of actin, paxillin, talin, vinculin, and the basal membrane relative to a gold substrate, and analyzed the changes caused by peroxide-induced senescence.

Senescence is the entry of proliferative cells into irreversible, permanent cell cycle arrest^18^. Senescent cells are significantly flatter and larger than proliferative cells and may have enlarged or multiple nuclei^19–21^. Senescent cells secrete the typical senescence-associated secretory phenotype (SASP), which leads to the secretion of proteases, interleukins, chemokines, growth factors, and matrix metalloproteases^22–24^. Cellular senescence is triggered by various stressors. For instance, telomere shortening and replicative senescence occur by repetitively passing cells^25,26^. Oxidative stress caused by reactive oxygen species (ROS) also leads to senescence^27^. An established method for triggering oxidative stress involves treatment with hydrogen peroxide (H_2_O_2_)^28–30^. Cells with H_2_O_2_-induced proliferative arrest show the same altered markers as replicative senescent cells and are thus an excellent in vitro model for aging research^31^. Cellular senescence plays an important role in aging and age-related diseases such as chronic kidney disease, neurodegenerative diseases, macular degeneration, and cancer^32–36^.

In this study, we aimed to determine whether and how focal adhesion complexes are restructured by oxidatively induced senescence. To determine the different heights of proteins in the focal adhesion complex, we used metal-induced energy transfer (MIET). With this tool, it is possible to detect the height of fluorescently labelled proteins above a metal layer with nanometer-scale accuracy. The axial resolution limit reaches 3 nm^37,38^. MIET works in a similar way to FRET^39^; however, while in FRET two fluorophores forming a FRET pair interact, in MIET, the acceptor molecule is replaced by a metal layer and strong optical near-field coupling of plasmons occurs. This results in a distance-dependent energy transfer between the metal layer and the donor, of up to 200 nm^37,38,40^. This distance is directly correlated with the fluorescence lifetime. MIET has previously been used to study the actin cytoskeleton, focal adhesion proteins, nucleus, and cell membrane^16,37,41,42^.

Although it is known that senescent cells form larger focal adhesions^43,44^, it is unclear whether the layered structure of focal adhesion complexes is affected by oxidative stress. To analyze this, we studied the focal adhesion complex of hydrogen peroxide-induced senescent cells with metal-induced energy transfer. Here, we show that senescence triggered by oxidative stress leads to restructuring of focal adhesion complexes. They exhibited a distinct pattern of cellular localization of talin and paxillin in senescent cells, which were located closer to the basal membrane than in non-senescent cells, whereas vinculin was detected at elevated heights. The loss of cytoskeletal tension via blocking of myosin II has a strong effect on the restructuring of the focal adhesion complex, decreasing the distance between the membrane and actin cytoskeleton, and increasing the distance of paxillin, vinculin, and talin relative to the membrane. RhoActivator leads to increased RhoA activity, resulting in the formation of actin stress fibers and focal adhesions, and increased myosin II activity. Treatment with RhoActivator elevated the actin cytoskeleton in wild-type cells, whereas the focal adhesion proteins paxillin, vinculin, and talin were localized closer to the membrane. In contrast, RhoActivator treatment in senescent cells localizes actin and paxillin closer to the membrane, whereas vinculin and talin are elevated and getting located very close to the actin cytoskeleton.

Blocking mechanosensitive ion channels reduces spreading in senescent cells, although the focal adhesion architecture of senescent cells is hardly affected by ion-channel blockage, as opposed to that of wild-type cells. This attenuated effect of blocking mechanosensitive channels in senescent cells suggests that cellular senescence reduces the membrane tension. A proteomics analysis also revealed that several proteins involved in mechanosensing, cytoskeleton, and ion channels were increased or decreased in abundance in H_2_O_2_ senescent cells.

## Results

In our study, we treated the 3T3 fibroblast cell line with 200 µM H_2_O_2_ for 2 h^30^ and induced senescence. Cellular intrinsic oxidation was measured using a Grx1-roGFP2-based sensor (roGFP) that monitors subcellular redox levels. The Grx1-roGFP2 sensor has a characteristic fluorescence profile according to the redox status of the roGFP cysteines, the oxidation of which leads to alternating peak intensities at 405 and 488 nm.

**Figure 1:**
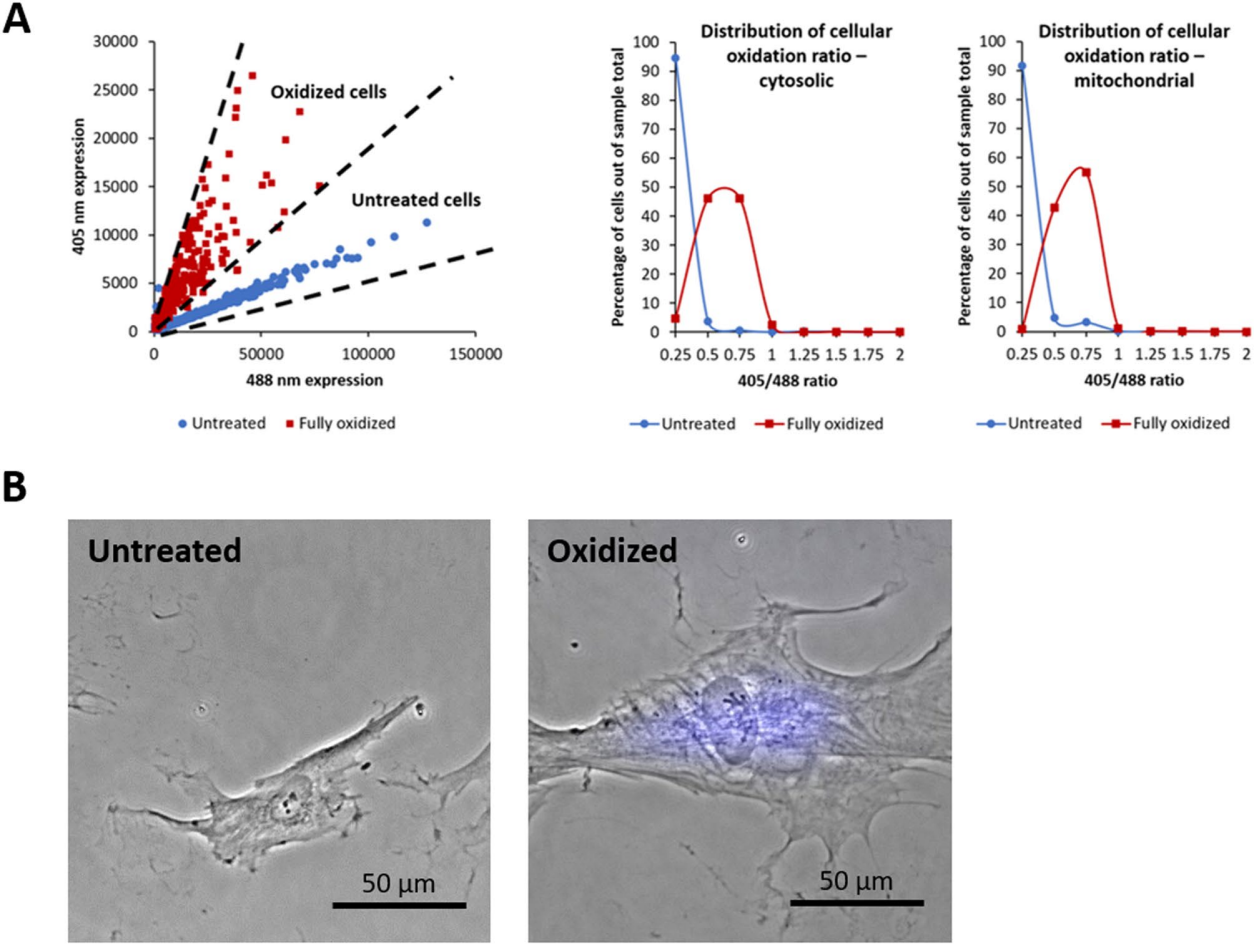
(**A**) Monitoring subcellular redox levels in the cytoplasm and mitochondria. (**B**) Senescence-associated β-galactosidase confirms that the H_2_O_2_-treated cell is senescent.

The roGFP2 sensor is expressed in the cytosol and can be targeted to different organelles (e.g., mitochondrial matrix) through the fusion of the defined signal sequence (e.g., *N.S*.ATP-9 in the mitochondrial roGFP2 sensor)^45^. After expression in the 3T3 fibroblast cells, the degree of oxidation was then assessed at the single-cell level for either cytosolic or mitochondrial sensors independently through flow cytometry fluorescent measurements in a ratio manner. Using fluorescence expression thresholds to define cells expressing the roGFP sensor and filtering out roGFP-negative cells, we measured the changes in the redox ratio (405 nm divided by 488 nm)^45^ of untreated cells and cells treated with H_2_O_2_ to achieve oxidation. As expected, peroxide treatment resulted in increased oxidation in both the cytosol and mitochondria, as reflected by the increased 405 nm/488 nm fluorescence ratio (Fig. 1A). H_2_O_2_-induced senescent cells showed senescence-associated β-galactosidase activity (Fig. 1B).

### Cell area

**Figure 2:**
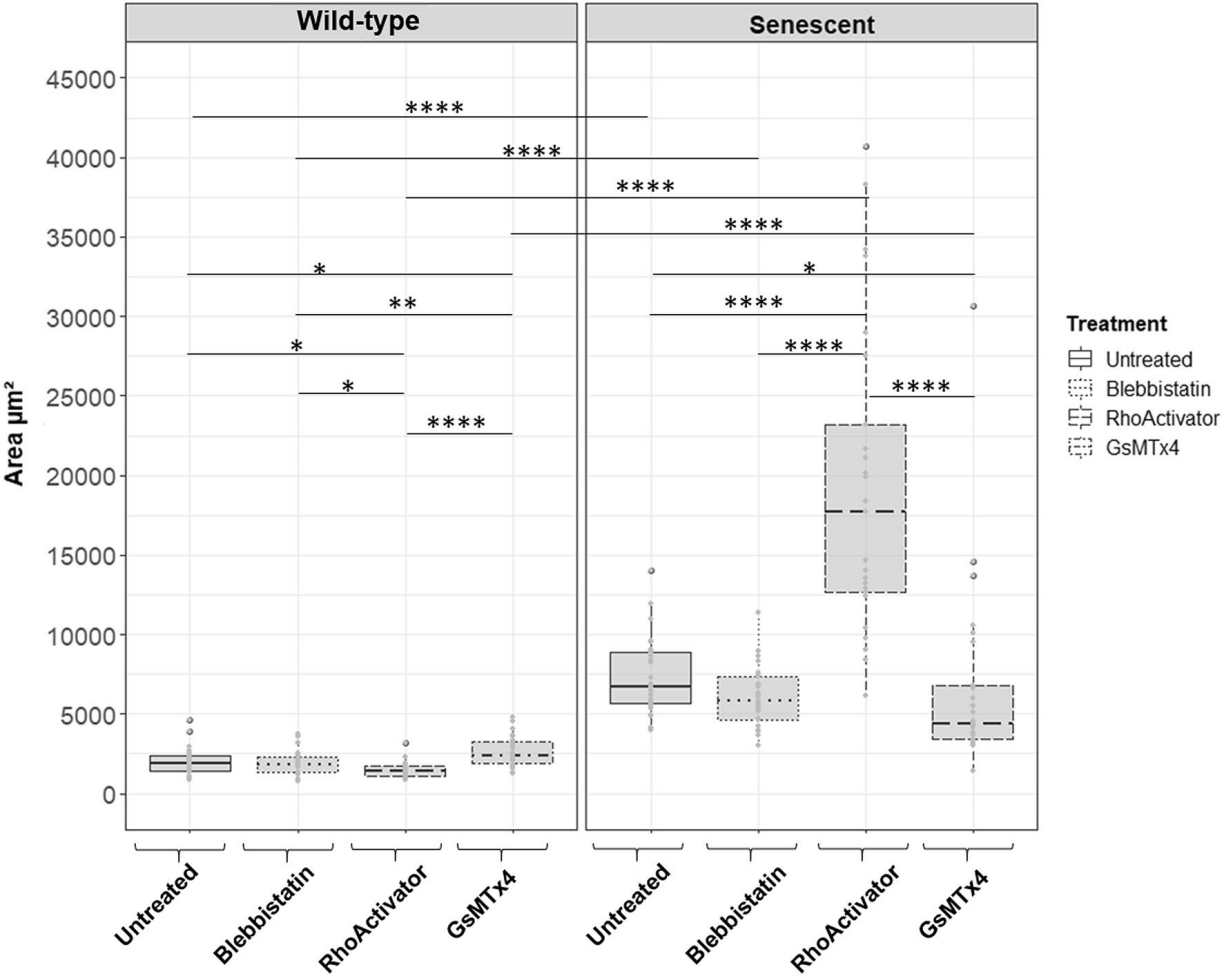
Area of wild-type and senescent fibroblasts (Untreated, Blebbistatin, RhoActivator, GsMTx4). Statistical test: Kruskal–Wallis Test with post hoc Dunn’s test, p > 0.05, *: p <  = 0.05, **: p <  = 0.01, ***: p <  = 0.001, ****: p <  = 0.0001, N = 25. The area of cells increased significantly with senescence. GsMTx4-treated senescent cells were smaller than the untreated and Blebbistatin-treated senescent cells. However, GsMTx4-treated wild-type cells covered a larger area than untreated, RhoActivator-treated, and Blebbistatin-treated wild-type cells. RhoActivator-treated cells showed the smallest area under all conditions in the wild-type cells and the largest area under all conditions in the senescent cells.

The projected cell area was significantly larger after the H_2_O_2_ treatment (Fig. 2). Both wild-type and senescent cells were treated with Blebbistatin, RhoActivator, or GsMTx4 after seeding. Blebbistatin is a myosin II inhibitor that affects cytoskeletal tension^46^, whereas RhoActivator increases the activity of RhoA. GsMTx4 inhibits mechanosensitive ion channels^47^. GsMTx4-treated wild-type cells have a larger area than Blebbistatin, RhoActivator, and untreated wild-type cells. Although RhoActivator-treated wild-type cells had the smallest area, by entering senescence, they showed the largest cell area under all conditions. In contrast, GsMTx4-treated senescent cells had a smaller area than untreated senescent cells, but a larger area than wild-type cells.

### Focal adhesion area

**Figure 3:**
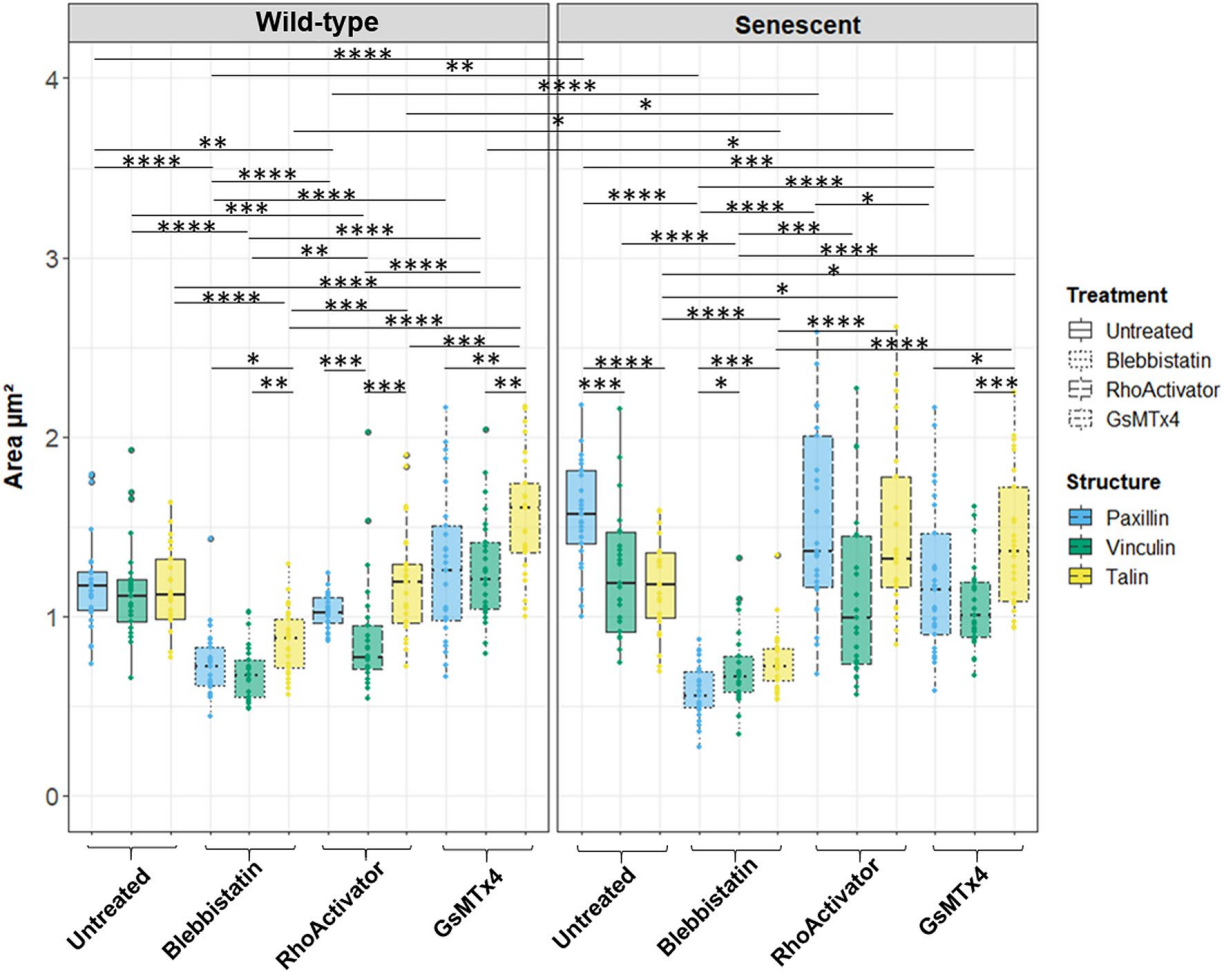
Focal adhesion protein (paxillin, talin, vinculin) areas of wild-type and senescent fibroblasts (Untreated, Blebbistatin, RhoActivator, GsMTx4). Statistical test: Kruskal–Wallis Test with post hoc Dunn’s test, p > 0.05, *: p <  = 0.05, **: p <  = 0.01, ***: p <  = 0.001, ****: p <  = 0.0001, N = 25. The area covered by paxillin increased strongly with senescence in the untreated and RhoActivator-treated cells. Among senescent cells, RhoActivator-treated cells also showed an increase in the area covered by talin and vinculin. In GsMTx4-treated cells, there was little difference in the area covered by vinculin between the wild-type and senescent cells. Blebbistatin-treated cells showed the smallest area of focal adhesions.

Fluorescent imaging of paxillin, talin, and vinculin in wild-type and hydrogen peroxide-treated cells revealed a strong effect on the focal adhesion area caused by both interference with cytoskeletal tension and H_2_O_2_-induced senescence (Fig. 3). The area covered by paxillin was significantly larger in senescent cells than that in wild-type cells. There was no change in the area covered by talin and a slight increase in the vinculin-covered area in senescent cells compared with that in wild-type cells. Vinculin and talin, which interact with paxillin in focal adhesions, cover an identical area to paxillin in wild-type, but not in senescent cells.

Blebbistatin-treated cells show a distinct change in morphology driven by the loss of tension in the cytoskeleton, both in wild-type and senescent cells. Focal adhesions hardly form at all, are concentrated mainly at the cell border, and are much smaller than in untreated and GsMTx4-treated cells, independent of H_2_O_2_ treatment. This indicates that tension is required for the maintenance of focal adhesions.

In senescent cells treated with RhoActivator, the area covered by paxillin and talin remained unchanged compared with wild-type senescence, whereas vinculin covered a smaller area. The focal adhesions of RhoActivator-treated cells differ greatly between wild-type and senescent cells; entry into senescence increases the area of paxillin, vinculin, and talin. Here, paxillin covered the largest area. Meanwhile, the inhibition of ion channels by GsMTx4 leads to opposite effects in wild-type and senescent cells. In wild-type cells, GsMTx4 significantly increased focal adhesion area, leading to the largest focal adhesions observed under all conditions, particularly for talin. In contrast, in senescent cells, the area covered by paxillin was strongly reduced after ion channel blockage.

To investigate how H_2_O_2_-induced senescence alters the focal adhesion structure, we used metal-induced energy transfer (MIET) to resolve the localization of select key proteins in the focal adhesion in the axial dimension with nanometer precision.

**Figure 4:**
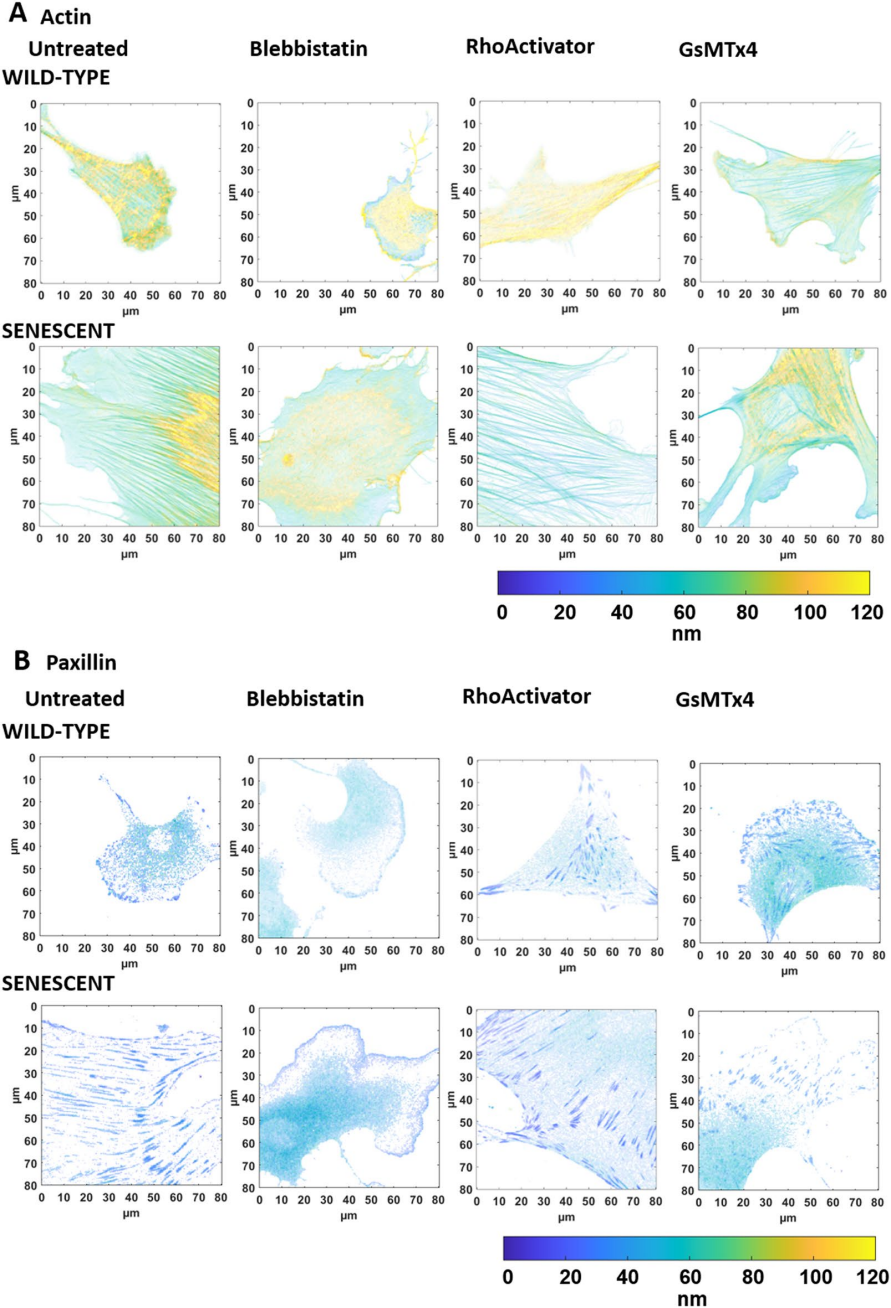
Intensity-weighted height images of wild-type and senescent fibroblasts (Untreated, Blebbistatin, RhoActivator, GsMTx4). (**A**) Actin Cytoskeleton: Senescent cells have a larger cell area than untreated cells. Treatment with Blebbistatin causes a loss of cellular structure, resulting in an actin cytoskeleton with fewer stress fibers. (**B**) Paxillin. Wild-type and senescent untreated, RhoActivator-treated, and GsMTx4-treated cells showed pronounced paxillin in focal adhesions throughout the cell. In Blebbistatin-treated cells, paxillin was concentrated at the cell edge and smaller.

**Figure 5:**
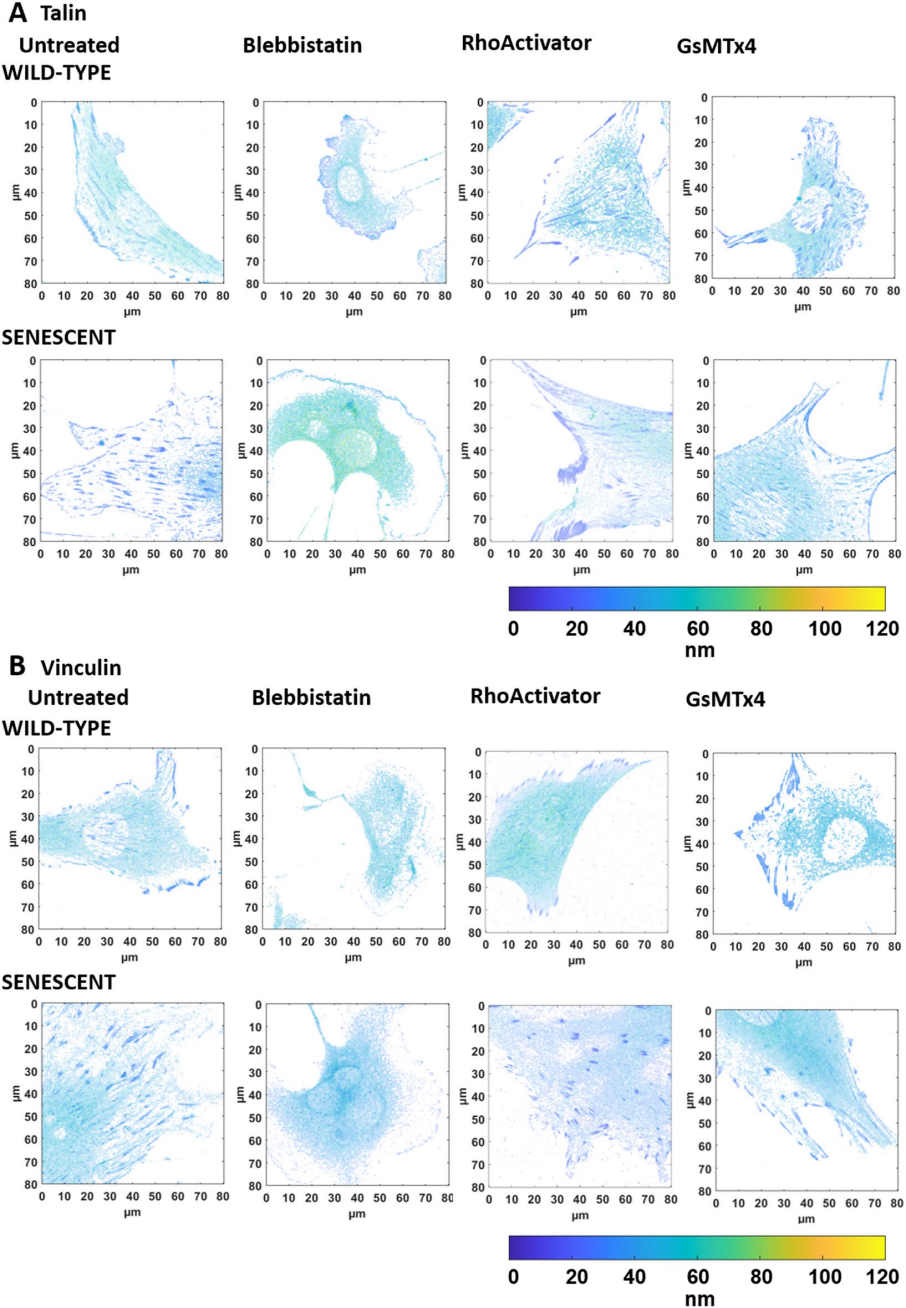
Intensity-weighted height images of wild-type and senescent fibroblasts (Untreated, Blebbistatin, RhoActivator, GsMTx4). (**A**) Talin, (**B**) Vinculin. Untreated, RhoActivator-treated, and GsMTx4-treated wild-type and senescent cells showed vinculin and talin throughout the cell. In Blebbistatin, these were concentrated at the cell edge and were smaller.

**Figure 6:**
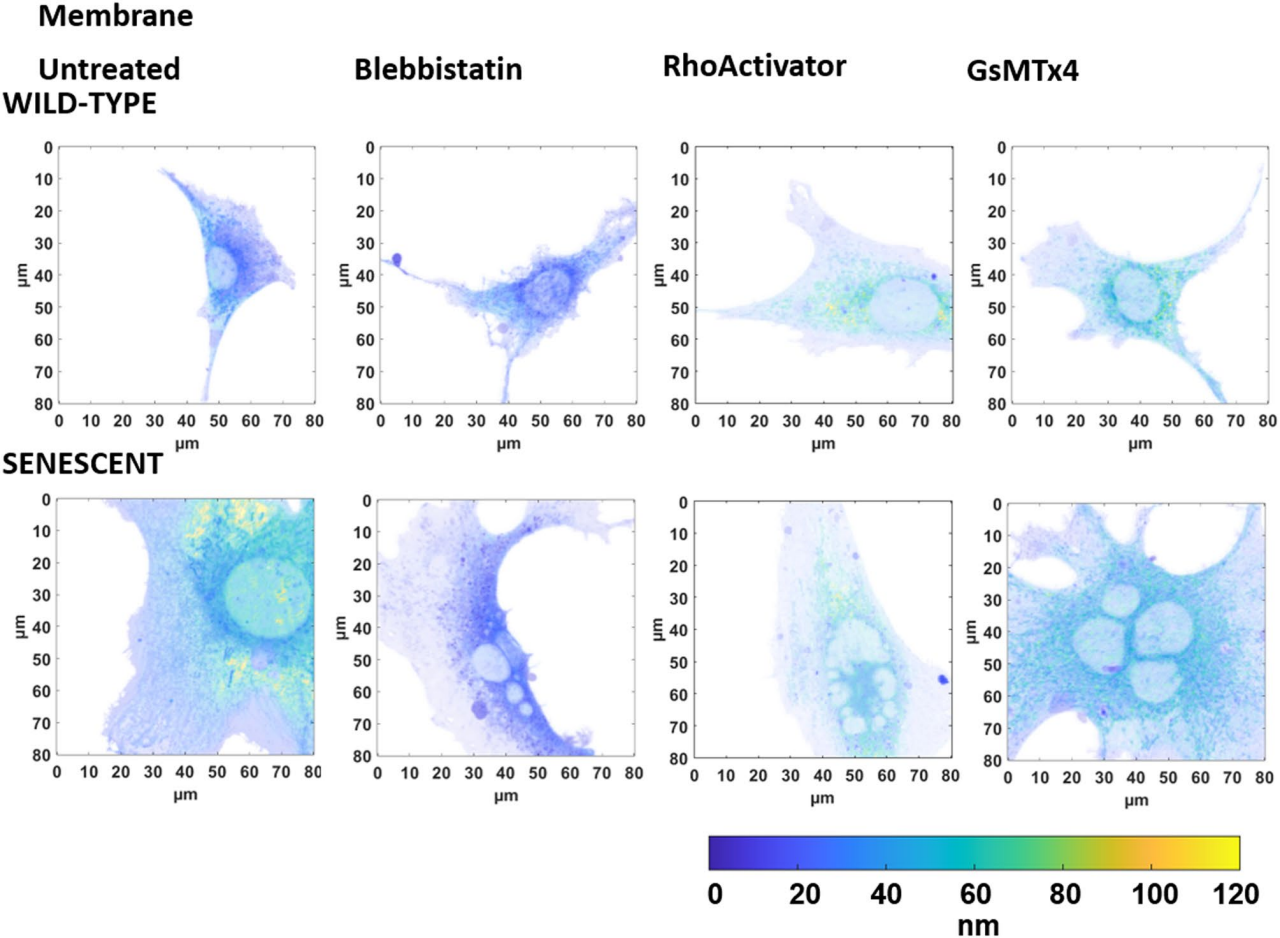
Intensity-weighted height images of wild-type and senescent fibroblasts (Untreated, Blebbistatin, RhoActivator, GsMTx4). Membrane. There was no visible change in the membrane following different treatments.

An illustration of the height-converted MIET image of representative cells is shown in Fig. 4 (actin and paxillin), Fig. 5 (talin and vinculin), and Fig. 6 (membrane).

**Figure 7:**
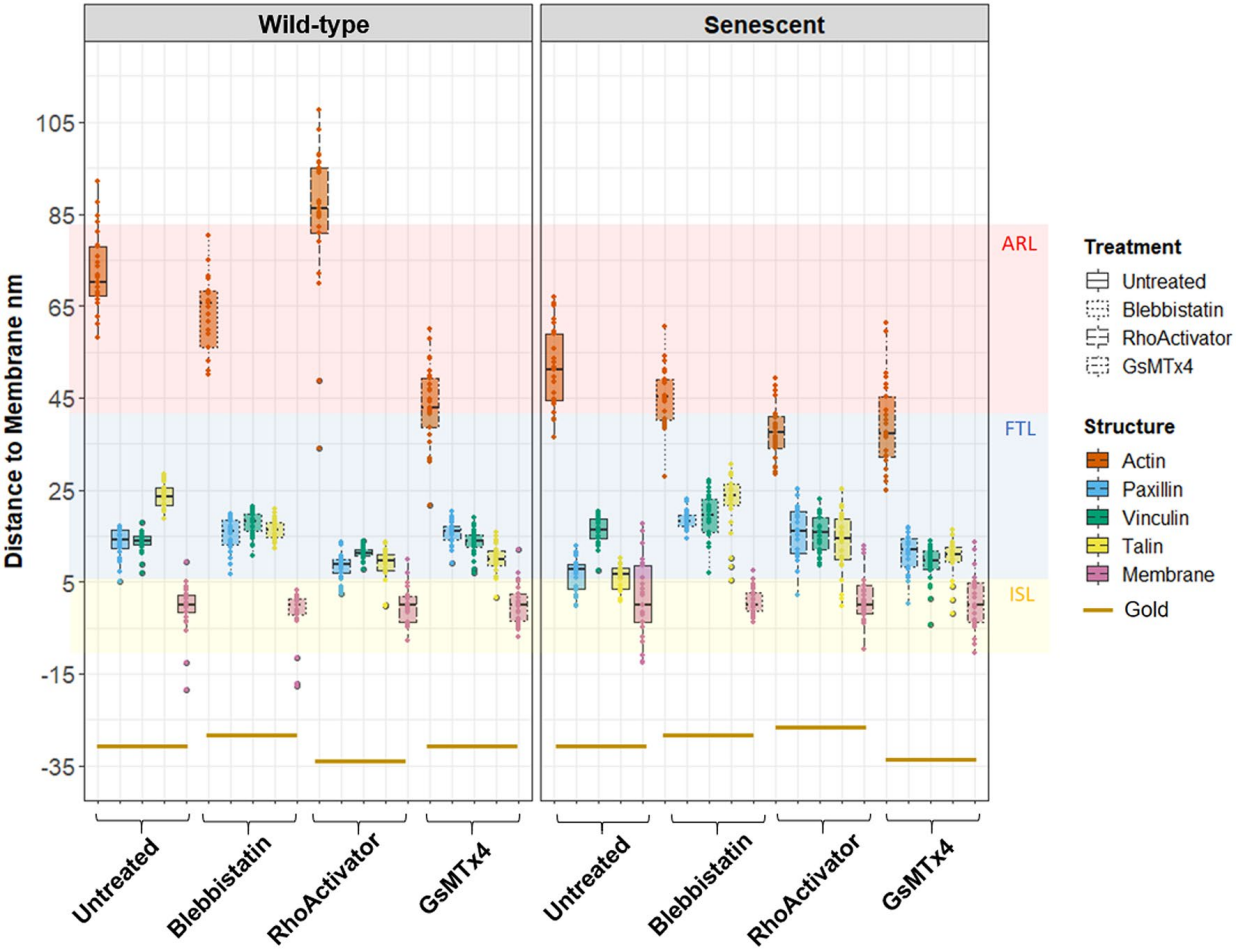
Distance to membrane height of wild-type and senescent fibroblasts (Untreated, Blebbistatin, RhoActivator, GsMTx4). Statistical test: Kruskal–Wallis Test with post hoc Dunn’s test, p > 0.05, * p <  = 0.05, ** p <  = 0.01, *** p <  = 0.001, **** p <  = 0.0001, N = 25 in n Fig. 8 and Supplementary Fig. 1S. ARL, actin regulatory layer; FTL: Force transduction layer; ISL: Integrin signaling layer. Untreated cells remodeled the focal adhesion complex during senescence. Actin, talin, and paxillin shifted downwards to the membrane. In Blebbistatin-treated wild-type and senescent cells, loss of tension results in a decrease in the actin cytoskeleton. The focal adhesion complex between wild-type and senescent Blebbistatin-treated cells hardly differs and is at approximately the same height. Increasing the activity of RhoA via the RhoActivator leads to a higher actin cytoskeleton in wild-type cells, whereas the actin cytoskeleton in senescent cells is significantly lower. Paxillin, vinculin, and talin heights were lower in RhoActivator-treated wild-type cells than in senescent cells. When the mechanosensitive ion channels were blocked by GsMTx4, actin was at the same height in both wild-type and senescent cells, but lower than that in untreated and Blebbistatin-treated cells. Small changes were observed in the focal adhesion complex.

**Figure 8:**
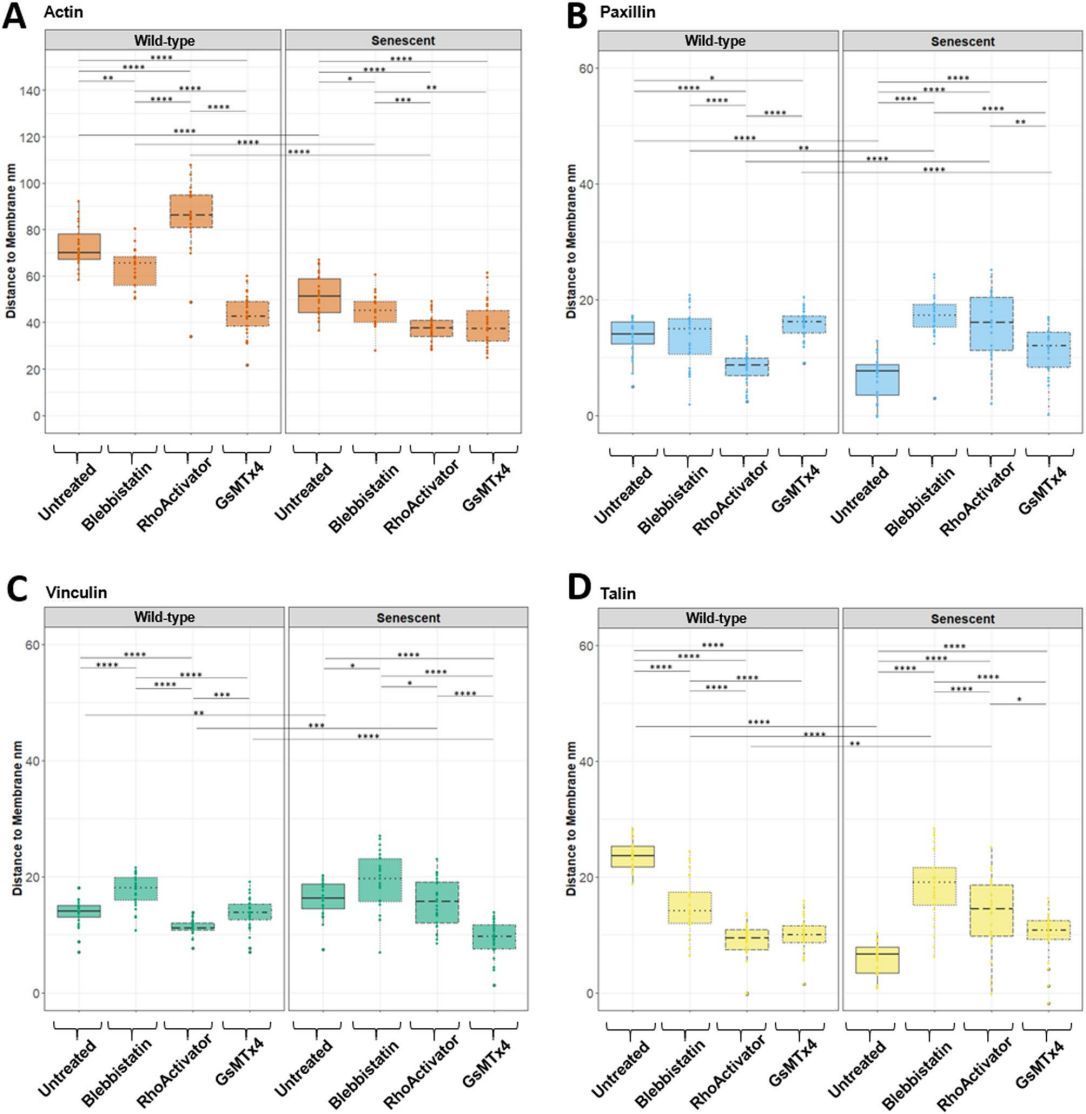
Distance to membrane height separated according to the proteins plotted in Fig. 7. (**A**) Actin. (**B**) Paxillin. (**C**) Vinculin. (**D**) Talin. Statistical test: Kruskal–Wallis Test with post hoc Dunn’s test, p > 0.05, *: p <  = 0.05, **: p <  = 0.01, ***: p <  = 0.001, ****: p <  = 0.0001, N = 25.

To thoroughly evaluate the changes in the height of the proteins, we calculated the median height per cell of each protein relative to the height of the membrane (Figs. 7 and 8). Hence, the membrane was used as a baseline to compare the heights of the focal adhesion proteins.

### Untreated

In untreated wild-type cells, paxillin and vinculin were at the same vertical distance from the membrane, whereas talin was at a significantly higher distance. The greatest distance from the membrane was observed for actin. In senescent cells, actin, paxillin, and talin were downshifted, while vinculin was slightly upshifted.

### Blebbistatin

In wild-type cells, loss of cytoskeletal tension via treatment with the myosin II inhibitor Blebbistatin strongly downshifts talin such that it localizes at the same height as paxillin, whereas vinculin is slightly upshifted. In senescent cells, loss of cytoskeletal tension strongly upshifts talin and paxillin, slightly upshifts vinculin, and slightly downshifts actin.

In a comparison of wild-type cells with senescent cells, both have a loss of tension by Blebbistatin treatment and localize actin significantly closer to the membrane, while vinculin and paxillin move away from it. In contrast, talin moves closer to the membrane in wild-type cells following tension loss, whereas in senescent cells, it moves away.

### RhoActivator

In wild-type cells, increased cytoskeletal tension due to the increased activity of RhoA resulted in a higher actin cytoskeleton and lower height of paxillin, vinculin, and talin. In senescent cells treated with RhoActivator, the height of the actin cytoskeleton was greatly reduced, and paxillin, vinculin, and talin were at the same height as vinculin in the untreated senescent cells.

### GsMTx4

Blocking the mechanosensitive ion channels with the GsMTx4 inhibitor led to a strong downshift of actin and talin in the wild-type, a significant upshift of paxillin, and no alteration of vinculin. In contrast, the changes in senescent cells were minor, with a small downshift of actin, an upshift of both paxillin and talin, and a downshift of vinculin. The strong reduction in effect magnitude in senescent cells may indicate that they have less membrane tension, thereby reducing mechanosensitive channel activity. Interestingly, treatment of both cell types with GsMTx4 nearly abolished the differences in focal adhesion architecture between the wild-type and senescent cells.

We followed this analysis by examining proteomic differences following H_2_O_2_ treatment in cells. Using mass spectrometry-based label-free quantitative proteomics, we identified 1065 proteins, with 116 proteins significantly more abundant in the control sample and 95 proteins significantly more abundant during H_2_O_2_ treatment (Fig. 2AS). Among these identified proteins, we found an expected enrichment for mitochondrial and stress response proteins during H_2_O_2_ treatment, as well as a moderate enrichment for calmodulin-binding functions (enrichment score 1.66, p = 8.10E−05) (Fig. 2AS). At the individual protein level, we identified several proteins, such as Anxa1, Cav1, Myo1c, Ehd2, Sptbn1, Sptan1, Thy1, Ppp3ca, and Cask, which were significantly more abundant during H_2_O_2_ treatment, as well as Pfn1, Pdlim1, Sh3kbp1, Mapre1, Itga6, and Rpsa, which were less abundant (Table 1). Several of these proteins are involved in the cytoskeleton, cell adhesion, mechanosensing, and calcium signaling.

**Table 1:** Proteins involved in mechanosensing are upregulated and downregulated in H_2_O_2_-treated senescent cells.

	Gene name	Protein name	Gene ontology
Up in H_2_O_2_	Anxa1	Annexin	Actin cytoskeleton organization, migration, cell shape
	Cav1	Caveolin-1	Focal Adhesion, cell migration, plasma membrane, calcium ion transport
	Myo1c	Unconventional myosin-Ic (Myosin I beta)	Actin cytoskeleton, plasma membrane, cell migration
	Ehd2	EH domain-containing protein 2	Cortical actin cytoskeleton organization
	Sptbn1	Spectrin beta chain, non-erythrocytic 1	Actin cytoskeleton organization
	Sptan1	Spectrin alpha chain, non-erythrocytic 1	Actin cytoskeleton
	Thy1	Thy-1 membrane glycoprotein (Thy-1 antigen)	Focal adhesion assembly, cytoskeleton organization, integrin-mediated signaling pathway, cell migration
	Ppp3ca	Protein Phosphatase 3 Catalytic Subunit Alpha	Calcium signaling
	Cask	Peripheral plasma membrane protein CASK (Calcium/calmodulin-dependent serine protein kinase)	Cell–matrix adhesion, calcium ion import
Down in H_2_O_2_	Pfn1	Profilin	Actin cytoskeleton organization
	Pdlim1	PDZ and LIM domain protein 1	Actin cytoskeleton organization, cell migration
	Sh3kbp1	SH3 domain-containing kinase-binding protein 1	Focal adhesion, actin filament organization
	Mapre1	Microtubule-associated protein RP/EB family member 1	Focal adhesion, cell migration, microtubule
	Itga6	Integrin subunit alpha 6	Cell adhesion, integrin-mediated signaling pathway
	Rpsa	Laminin receptor	Cell surface receptor for laminin, cell adhesion signaling

Gene ontology was based on the UniProt server.

## Discussion

Our results clearly show a strong effect of senescence on focal adhesion structure. These changes in focal adhesions may contribute to the strong phenotypic alterations and different behaviors of senescent cells. The proteins in the focal adhesion complex are organized in different layers: the integrin signaling layer (approximately 0–10 nm above the membrane), the force transduction layer (approximately 10–40 above the membrane), and the actin regulatory layer (approximately 40–80 nm above the membrane). The definition of this layer varies slightly^6,7^. In comparison, Kanchanawong et al.^6^ measured the actin distance relative to the membrane between 50 and 80 nm, Case et al. of 60 nm to the membrane^7^, and ourselves of 70 nm to the membrane. Kanchanawong et al.^6^ measured a range of 10–30 nm above the membrane for paxillin and 20–40 nm above the membrane for vinculin. Case et al. obtained values of approximately 41 nm above the membrane for vinculin and 25 nm above the membrane for paxillin. Chizhik et al.^42^ also measured focal adhesions via MIET and obtained values of approximately 20 nm for vinculin and 17 nm for paxillin. These results also indicated a similar location of vinculin and paxillin in the focal adhesion of untreated wild-type cells, in accordance with our results. Kanchanawong et al.^6^ measured 20–30 nm above the membrane for the talin N-terminus and 40–80 nm above the membrane for the talin C-terminus. This comparison between the different measurements shows a consensus focal adhesion architecture, despite the analysis of different cell types.

**Figure 9:**
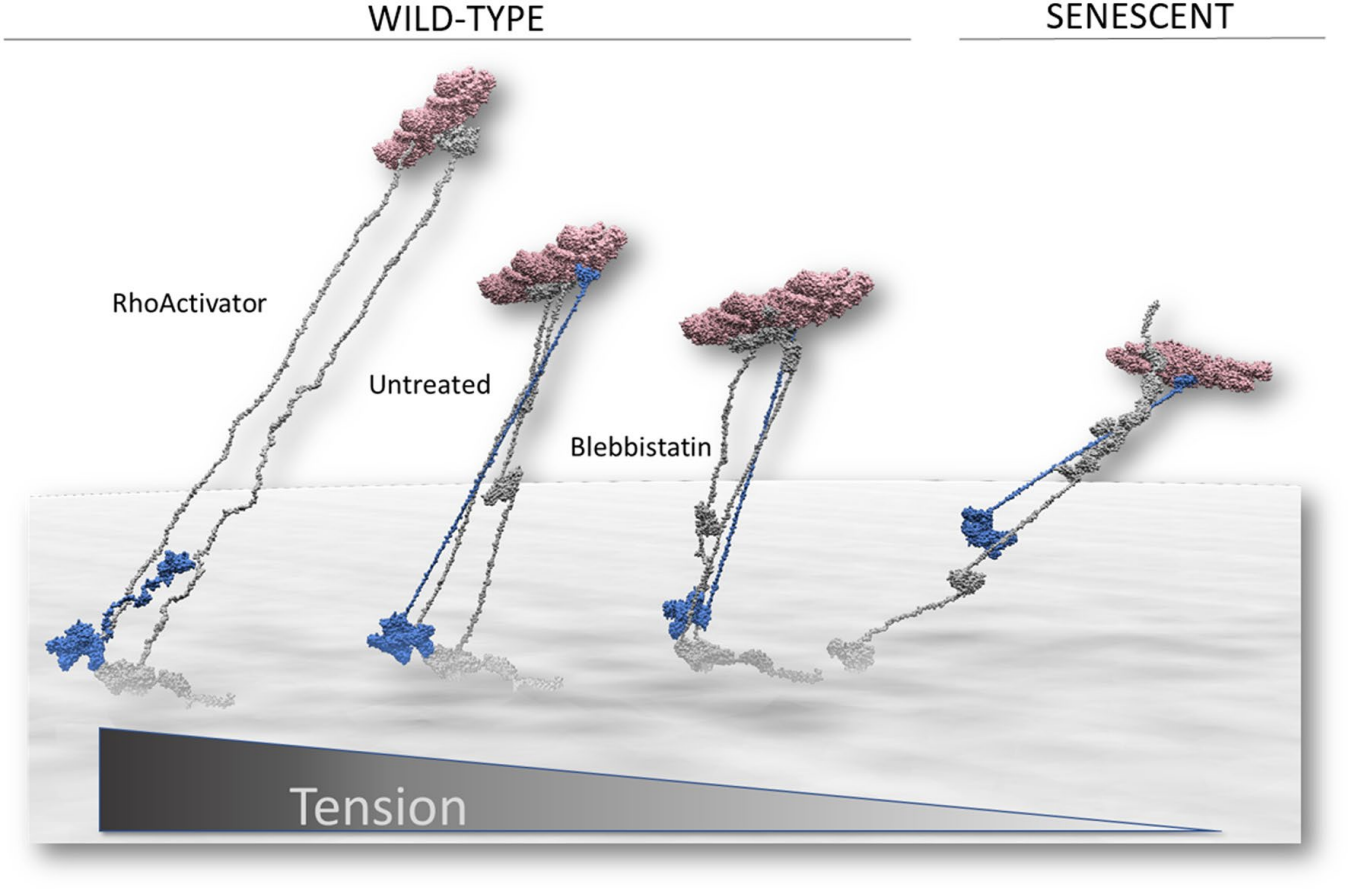
Models of the ternary talin (gray)-vinculin(blue) -actin (red) complex. With decreasing tension, the distance between actin and the membrane decreased. This is structurally compatible with an accompanying increase of folded talin rod domains. The relative orientation of vinculin and talin in our measurements suggests the engagement of talins IBS2 in wild-type cells but not in senescent cells.

In focal adhesion, many proteins adopt a variety of strain-dependent conformations, leading to a large number of possible structures. Therefore, the small number of structural restraints obtained from our measurements did not allow the construction of a unique and unambiguous structural model. However, our data can serve as scaling guidelines for examining the consistency of spatial restraints from protein structures with our data. Therefore, we created structural models based on the crystal structures of vinculin and talin and known biochemical and biomechanical data, along with our distance measurements (Figs. 9 and 3S). Talin organizes the nanoscale architecture of focal adhesions^48^. It is a long and flexible protein consisting of an N-terminal FERM domain, 11 rod domains, and a dimerization helix, with each domain containing multiple binding sites. These include two integrin-binding sites (IBS1 and IBS2, one in the N-terminal FERM domain and one in the rod 11 domain), three actin-binding sites (ABS1, ABS2, and ABS3) located in the FERM domain (ABS1), in the R13 domain (ABS3), and cooperately on R8 and R4 (ABS2)), and 11 hidden vinculin-binding sites in different rod domains. These become exposed when talin is subjected to strain^49^. This domain structure, together with the measured distances to the membrane, guided our structure-based analysis of the MIET results. First, we constructed a model of the talin-vinculin-actin complex in wild-type cells. The membrane was located relative to the talin FERM domain according to the Orientation of Proteins in Membrane (OPM) database^50^, using the structure of the integrin talin complex (PDB:3g9w^51^). Superimposing a model of a linear full-length talin (thankfully obtained by Benjamin Goult) on the FERM domain in an orientation approximately perpendicular to the membrane led to the positioning of the most membrane-proximal vinculin-binding site in talin rod 1 above the antibody epitope on talin, which contradicts our measurements. One intriguing possibility for resolving this contradiction is to position talin such that both IBS1 and IBS2 engage in integrin binding. In the absence of an experimental structure of IBS2 in complex with integrin, we manually located rod 11 close to the membrane to test this possibility. This locates rod domain 10 (R10) (which contains a vinculin-binding site) close to the membrane, at a distance consistent with our measurements. In support of this model, the engagement of IBS2 has been shown to be crucial for linking the actin cytoskeleton to integrins in mature focal adhesions^52,53^. Such positioning of both IBS1 and IBS2 additionally locates ABS1 and ABS3 close to the membrane at distances incompatible with our measurements. However, ABS2 can be positioned at a distance in accordance with our measurements provided that some rod domains unfold. This implies that ABS2 is essential for force transmission in mature focal adhesion. Indeed, biochemical studies have shown that ABS2 is critical for focal adhesion maturation^54^. In addition, FRET measurements using tension sensors incorporated into talin demonstrated that the primary actin-binding site responsible for force transmission is ABS2^55^. The unfolding of talin rod domains has been extensively demonstrated. Talin has been shown to extend to hundreds of nanometers under moderate strain^56^. The talin helix bundles in the talin rod can unfold into an open state under mechanical stress, leading to significant lengthening, as observed in steered molecular dynamics simulations and experiments^57^. Vinculin binds up to 11 helices in the talin rod only after unfolding of these rod domains^58^, even contracting already unfolded talin segments^59^ and preventing rapid refolding. Thus, the helix bundles in the talin rod represent force-dependent binary-length switches, as discussed in detail by Goult et al.^60^. Located between integrin binding site 1 (IBS1), which locks the talin FERM domain to the membrane, and ABS2 are the rod domains R1-R3, and between ABS2 and IBS2 are R10 and R9, and the two C-terminal helices of R7. Although R8 has been shown to be weaker than R7^56^, once bound to actin, it may be strengthened and protected against unfolding. Unfolded R3 (in line with earlier observations^61^), R10 (which is weaker than R9^56^), and the two C-terminal helices of R7 locate actin, vinculin, and talin at heights consistent with our measurements. In this model, the vinculin head binds to the vinculin-binding site of R10, and the tail domain can bind actin in a strained conformation, strengthening the complex and forming a catch bond^62^. Hence, a structural model in agreement with our experiments presented here and with earlier biophysical and biochemical results involves the engagement of IBS1, IBS2 and ABS2, and a vinculin link between the VBS in talin R10 and actin.

Treatment with RhoActivator increases cytoskeletal tension. Increased tension leads to the additional unfolding of the talin rod domains. The observed elevation of actin required the unfolding of R9 and R1/R2 in our model. However, the observed actin distance indicated a more tilted conformation of talin with respect to the membrane. This puts both talin and vinculin closer to the membrane compared to untreated cells, which is in agreement with our measurements. However, vinculin could not span the distance between the vinculin-binding site in R10 and actin at an elevation of 90 nm in our model. Hence, either vinculin unfolds, as suggested by steered molecular dynamics simulations^63^, or the tail domain binds other partners, such as paxillin^64^.

Treatment with Blebbistatin reduces cytoskeletal tension. This is in structural agreement with the refolding of the two helices in R7, pulling actin closer to the membrane, in agreement with our measurements.

Piezo blockage in wild-type cells resulted in structures similar to those observed in senescent cells. Piezo is a membrane tension-activated ion channel. Therefore, the similarity between senescent cells and GsMTx4-treated cells indicated a loss of membrane tension in senescent cells. Indeed, strain-dependent Piezo-mediated Ca^2+^ influx is responsible for integrin turnover caused by calpain cleavage and focal adhesion disassembly^65^. Therefore, the larger focal adhesion area in senescent cells supports this loss of membrane tension and the accompanying loss of Piezo-mediated Ca^2+^ influx, preventing calpain cleavage. Interestingly, the structure of focal adhesions in senescent cells was drastically different from that in wild-type cells. Actin is closer to the membrane and, most importantly, vinculin is located above talin. This finding supports a model in which IBS2 is not involved. Decreasing the tilt angle of talin and using ABS3 locates actin, vinculin, and talin to the height measured in this study in senescent cells. This model is reminiscent of the model proposed for early talin activation during focal adhesion maturation^54^. Hence, our results suggest a dramatic loss of tension per talin molecule in senescent cells, which results in a focal adhesion structure in which only IBS1 is engaged and only R3 is unfolded. Because protein unfolding is crucial for mechanosignaling^66^, our results may support disturbed mechanotransduction in senescent cells. The effects of Piezo blockage in senescent cells can be understood as an even stronger reduction in tension per molecule, whereas RhoActivator treatment led to a partial rescue of the wild-type structure; paxillin, vinculin, and talin were at heights comparable to those in untreated wild-type cells. This indicates that additional cytoskeletal tension enables cells to re-stabilize focal adhesions. However, differences in the expression of actin-organizing proteins lead to a failure of complete rescue of the cells, leading to further imbalances in tension and drastically increased cell and focal adhesions in senescent cells^44^. Our proteomic data showed that caveolin and calmodulin are upregulated in senescent cells. Cho et al.^43^ also observed that caveolin is upregulated in senescent cells and affects focal adhesion kinase, as it binds to integrins in the membrane. In that study, talin and vinculin were not investigated, but talin could bind to focal adhesion kinase. The increased release of calmodulin suggests that the CaMCKII pathway, which is dependent on integrins, is also involved^67,68^. This also indicates a larger adhesion complex, which reduces tension per molecule. Calmodulin activates myosin light-chain kinase, which increases cell contraction^69^. However, the enlargement of focal adhesions counteracts this increase, effectively decreasing the strain per molecule.

Blebbistatin treatment results in senescent cells are somewhat puzzling, since the distances of Blebbistatin-treated senescent cells closely resemble the distances of Blebbistatin-treated wild-type cells. This may be partially caused by the very high background in these measurements, as Blebbistatin dissolves focal adhesions. Potentially, a subset of focal adhesions is unaffected by senescence, and these may be more resistant to Blebbistatin treatment, and therefore, potentially dominate our measurements.

Here, we only capture a static picture of an average of focal adhesions, disregarding dynamic aspects. However, in force-transmitting junctions between integrins and the cytoskeleton, F-actin flows across focal adhesions^7^. In focal adhesions (FAs) near cell edges, actin flows rearward over immobile integrins, whereas talin and vinculin move rearward at intermediate velocities. The different flow velocities of integrins, vinculin, talin, and actin suggest that the binding between integrin and F-actin mediated by talin and vinculin is dynamic, with the rapid association and dissociation of vinculin and talin^55^. Therefore, it is possible that only for untreated wild-type cells, focal adhesion homeostasis is so accurately balanced that the majority of talin is engaged in actin binding, while both increase and loss of tension leads to an increase in unbound talin. It is well known that talin binds to the membrane, and recent results stress the importance of the talin-membrane interaction of focal adhesion assembly^70^. This concept of dynamic binding and unbinding of talin and vinculin in focal adhesions, with a pool of membrane-bound proteins that are poised to engage in focal adhesion binding, would also explain the close positioning of vinculin to the membrane in our experiments and agree with the dynamic nature of vinculin and talin binding in focal adhesions. Furthermore, recent theoretical results have shown that a pool of unbound proteins is required for focal adhesion growth and strengthening^71^.

Again, we would like to stress that the large number of possible conformations and small number of spatial restraints preclude us from unambiguously constructing unique models. Therefore, the models presented here need to be adapted if more restraints become available.

Previously, we demonstrated a sophisticated balance between adhesion tension and cytoskeletal tension^16^. An imbalance between the two different tensions causes accommodation of the actin cytoskeleton and the focal adhesion complex. An increase in cytoskeletal tension results in an elevated actin cytoskeleton, whereas an increase in adhesion tension results in a lowered actin cytoskeleton^16^. This may explain the lowered actin positioning in RhoActivator-treated senescent cells; the treatment leads to an increase in focal adhesion area, which causes an increase in adhesion tension, resulting in a large increase in cell area and a structural downshift of actin. We have also shown in another study that a higher adhesion tension leads to an upshift in vinculin^72^. Senescent cells showed a significantly lower cytoskeleton, an upshift of vinculin, and a downshift of talin, suggesting that in senescent cells, a tension imbalance exists, and adhesion tension dominates, which is expressed in larger focal adhesions and a larger cell area with actin being located closer to the membrane. This increase may be caused by reduced activity of the Piezo channels caused by a loss of tension, since Piezo channels are important for calcium influx, causing calpain activation. Activated calpain cleaves talin and reduces the focal adhesion area, a mechanism that is potentially disturbed during senescence. Indeed, the inhibition of mechanosensitive ion channels, and thus the lowered influx of calcium, has a less dramatic effect on senescent cells than on wild-type cells. Actin in senescent and non-senescent cells hardly differed under the influence of GsMTx4 (43 ± 8 nm). In senescent cells, focal adhesion proteins in the integrin signaling layer accumulate close to each other (approximately 10 nm above the membrane). Vinculin is located closer to the membrane (10 ± 4 nm) than in the wild-type cells (14 ± 3 nm). Instead of an increase in the area of wild-type cells, there was less enlargement of senescent cells treated with GsMTx4 than in untreated or Blebbistatin-treated cells. Piezo1, which is inhibited by GsMTx4, is an ion channel that is activated by mechanical force and provides calcium influx into the cell^73,74^. Jetta et al.^75^ have shown that Piezo1 is involved in cell spreading events.

This study was performed using 3T3 mouse fibroblasts, which are a cell line of fibroblasts and not primary cells; therefore, they may behave differently in terms of mechanotransduction due to differences in cell adhesion complexes and cytoskeletal organization^76,77^. It would be interesting to test whether there is a different behavior in primary cells. It would also be interesting to investigate how the focal adhesion complex dynamically changes during senescence, as the temporal component was neglected in this study.

The broad distributions of distances observed in our study were mostly due to cell-to-cell variations. Chizhik et al.^37^ made great efforts to quantify the errors from MIET measurements, and they convincingly showed a measurement error of down to 3 nm depending on the number of photons. Therefore, MIET is much more accurate than the variations in our data because of cell-to-cell and height variations within the cell. The variations within a cell are greater than the intrinsic errors in the measurements, and the uncertainty in the positioning of the antibody epitopes leads to greater errors than the intrinsic measurement errors.

Other studies have shown that the integrin β3 subunit is a regulator of senescence^78,79^. Cells expressing only β3 integrins showed enlarged focal adhesions with reduced tension, similar to what we observed in senescent cells. Blocking β3 integrin signaling has been shown to inhibit senescence^78^. Analyzing these exciting correlations mechanistically in further detail in future studies will be fruitful.

Focal adhesions are the key protein complexes involved in mechanosensing. The intricate focal adhesion remodeling in senescent cells observed in this study suggests that mechanosensing is disturbed under these conditions. Further studies are needed to study altered mechanosensing in senescent cells in more detail.

## Methods

### Coating for MIET measurements

Glass coverslips (Paul-Marienfeld, Lauda-Königshofen, Germany) were silanised with (3-Mercaptopropyl) trimethoxysilane (MPTMS) (Sigma-Aldrich, Darmstadt, Germany) via evaporation and coated with 20 nm gold (Kurt-Lesker, Dresden, Germany) as previously described^80^. The gold coverslips were treated with 4 mg/mL dithiobis (succinimidyl propionate) (DSP) (Thermo-Fisher, Waltham, MA, USA) crosslinker for 30 min, followed by a 2 h coating with fibronectin (Sigma-Aldrich, Darmstadt, Germany), which specifically binds integrins via the RGD sequence^81^.

### Cell culture, drug treatment and immunofluorescence

NIH 3T3 mouse fibroblasts (ATCC® CRL-1658™, Manassas, Virginia, USA) were grown in Dulbecco's Modified Eagle Medium. The medium was supplemented with 10% fetal bovine serum (Bio&Sell, Feucht, Germany), and 1% antibiotic/antifungal solution (GE Healthcare Hyclone, Chicago, Illinois, USA). Before the cells were seeded on fibronectin-coated coverslips, they were detached using trypsin/EDTA (Biowest, Nuaillé, France) for 3 min. To induce hydrogen peroxide-induced senescence in cells, 3T3 cells were incubated for 2 h in 200 µM hydrogen peroxide (Sigma-Aldrich, Darmstadt, Germany). The cells were then incubated in the medium for 72 h^29,30^. Senescence was verified via senescence-associated beta-galactosidase staining using the CellEvent Senescence Green Detection Kit (ThermoFisher, Waltham, MA, USA).

Three different treatments were used: Blebbistatin samples were incubated with 50 µM Blebbistatin (Sigma-Aldrich, Darmstadt, Germany) for 40 min, RhoActivator-treated cells were treated for 3 h with 1 µg/µL Rho Activator II (Cytoskeleton, Denver, CO, USA), and GsMTx4 (Abcam, Cambridge, United Kingdom) samples were treated with 3 µM GsMTx4 for 3 h^82^. Before treatment, the cells were incubated overnight on coverslips.

Different structures were stained for the experiments. Cell Mask Orange (C10045, Thermo Fisher Scientific, Waltham, Massachusetts, USA) at a concentration of 5 µg/mL for 5 min followed by fixation with 4% warm paraformaldehyde was used to stain the membrane. Cells for immunostaining of F-actin were fixed in 4% warm paraformaldehyde (Polysciences, Warrington, USA) for 10 min, followed by permeabilization with 0.8% Triton X-100 (Sigma-Aldrich, Darmstadt, Germany) for 10 min. Cells to be stained with paxillin were treated with 4% warm paraformaldehyde for 10 min, followed by permeabilization with 0.1% Triton X-100 (Sigma-Aldrich, Darmstadt, Germany) for 5 min. For vinculin and talin staining, cells were fixed with ice-cold methanol (Sigma-Aldrich, Darmstadt, Germany) for 3 min. All the samples were incubated with 3% BSA (Sigma-Aldrich, Darmstadt, Germany) for 1 h. Primary antibodies were then used for paxillin (1:500, 610052, BD Biosciences, Franklin Lakes, New Jersey, USA), talin (1:1000, T3287, Sigma-Aldrich, Darmstadt, Germany), and vinculin (1:40, V4505, Sigma-Aldrich, Darmstadt, Germany) for 1 h, followed by incubation with anti-mouse secondary antibody Alexa568 antibody (1:500, A10037, Thermo Fisher Scientific, Waltham, Massachusetts, USA) for 1 h. Actin cytoskeleton staining was performed overnight with Alexa Fluor 568 phalloidin (1:100, A12380; Thermo Fisher Scientific, Waltham, Massachusetts, USA). All steps except overnight staining at 4 °C were performed at room temperature.

### FLIM setup and evaluation

For fluorescence lifetime imaging microscopy (FLIM), a MicroTime 200 (PicoQuant, Berlin, Germany) with an inverted Olympus IX73 microscope (Hamburg, Germany) was used. The setup contained a pulsed diode laser (LDH-D-TA-560, pulse width ~ 56 ps, repetition rate 40 MHz, wavelength 560 nm) and a high-numerical-aperture objective (60 × 1.2 UPlanSApo, Superapochromat, water immersion, WD = 0.28 mm). For data acquisition, a picosecond event timer HydraHarp 400 TCSPC module (PicoQuant, Berlin, Germany) was used. The measurements were recorded using SymPhoTime 64 software (PicoQuant, Berlin, Germany) and analyzed using a multi-exponential deconvolution fit. Further details have been previously described^16^.

### MIET analysis

In the MIET GUI program^37,38^ of the Enderlein Group (University of Göttingen, Germany), a calibration curve is created based on the refractive indices of the system to convert the measured lifetime into height data. The base of the sample was glass (refractive index n = 1.52), followed by silane (refractive index n = 1.443^83^) and 20 nm gold, which has a wavelength-dependent refractive index. On top of the gold is the cell (refractive index n = 1.3623^84^) and the mounting medium. The height values were then calculated using custom-written MATLAB code. For analysis of the height and images of the focal adhesions, the background was filtered. The cell area and focal adhesion area were analyzed using custom-written MATLAB code.

### FACS

3T3 fibroblast cells were transfected with a Grx1-roGFP2-based sensor (roGFP) with either cytosolic or mitochondrial localization, which monitors subcellular redox levels based on the ratio of fluorescence at 405 and 488 nm. Fluorescence was then measured using FACS with excitation at 405 and 488 nm. We manually defined roGFP-positive cells based on the fluorescence expression thresholds for each excitation (greater than 5 × 10^2^ at 488 nm and 2 × 10^2^ at 405 nm), for which we measured the redox ratio (405 nm divided by 488 nm)^45^ of untreated cells and cells treated with 200 µM H_2_O_2_ to achieve oxidation. The redox ratio of the oxidized cells was accordingly higher in the treated sample (Fig. 1), as determined using both cytosolic and mitochondrial sensors (measured independently). Data were extracted from. fcs files using FCS extract. The roGFP 405/488 ratio was then calculated for cells expressing the roGFP sensor and the ratios were distributed as a histogram according to a bin size of 0.25.

### Proteomics

Protein lysates of the cells from three biological replicates were prepared using RIPA Buffer (Thermo Fisher Scientific, Waltham, Massachusetts, USA). In-gel digestion of the proteins was performed by loading the protein lysates onto a gel and separating them at 75 V for 15–30 min. The gel was stained with Coomassie Blue (Thermo Fisher Scientific, Waltham, Massachusetts, USA) and the band was cut out. Excised gel bands were prepared for in-gel digestion of proteins, according to a previously described protocol. Gel bands were destained with 1 mL of buffer containing 50% 25 mM Tris HCl pH 8 (“Tris Solution”) and 50% ACN (HPLC-grade) for 15 min at 50 °C under agitation, repeated 3–4 times until all samples were fully destained. The liquid was discarded and the gels were resuspended in 0.5 mL ACN 100%, vortexed, and incubated for 5 min at room temperature before being discarded. The gels were incubated with Tris Solution containing 10 mM DTT for 30 min at 37 °C under agitation. Gel samples were then washed once with DDW (0.5 mL) before a 5-min incubation with 100% ACN (after vortexing). The gels were then incubated in the dark in 400 μL of Tris Solution with iodoacetamide (IAM) at a final concentration of 55 mM for 30 min at 37 °C under agitation. The liquid was again removed and the gels were washed with DDW and 100% ACN for 5 min. Gels were then incubated overnight at 37 °C in 400 μL Tris Solution with 0.5 μg Trypsin (Promega) with end-over-end rotation. The next morning, samples were diluted with 400 μL ACN 100% with formic acid (FA) at a final concentration of 2.5% and incubated for 30 min at 50 °C under agitation. Liquid was collected and samples were concentrated using SpeedVac (30 min at 45 °C), cleared of gel fragments (centrifugation at 12,000 × g for 10 min), loaded onto prepared C18 Stage Tips, washed, and eluted (as described in^85^).

Eluted peptides were then injected into a 100 μm i.d. × 2 cm Nano Trap Column packed with Acclaim PepMap100 C18 5 μm 100 Å (Thermo Scientific) for 8 min at a flow rate of 5 μL/min. Peptides were then separated on a C18 reverse-phase column coupled to a EASY-spray Nano electrospray column (PepMap, 75 mm × 25 cm, Thermo Scientific) at a flow rate of 300 nL/min, using a Dionex Nano-HPLC system (Thermo Scientific) online coupled to an Orbitrap mass spectrometer Q Exactive HF (Thermo Scientific). Peptide separation was performed through a linear gradient of ACN buffer (80% ACN with 0.1% FA) applied to the column at 300 nL/min, beginning with 1% ACN for 35 min. Samples were then injected and the flow rate was applied at 37 min: from 1 to 4% at 40 min at 300 nL/min, then 200 nL/min for 45 min, 4–28% at 150 nL/min at 135 min, 28–50% at 150 nL/min at 152 min, 50% at 200 nL/min at 157 min, 50–80% at 167 min, and held at 80% 200 nL/min until 179 min. The Q Exactive HF was operated in data-dependent mode. The survey scan range was to 300–1650 m/z at a resolution of 60,000. The isolation window was 1.6 m/z, and the dynamic exclusion was set to 20 s. The maximum injection time was set to 20 ms with an AGC target of 3 × 106 for full MS, while MS/MS was set at 25 ms and 10^5^, respectively. Data were acquired using Xcalibur software (Thermo Scientific). The columns were washed with 80% ACN and 0.1% FA for 55 min between each sample. Data were analyzed using MaxQuant software (version 1.5.3.30) and Perseus^86,87^, using default settings and an FDR < 0.05. Only proteins with three valid LFQ values (from three replicates) in at least one group were considered for statistical analysis. If needed, missing values (in the other group) were replaced by imputation with a fixed value of 22 after log2 transformation of LFQ intensities. Functional enrichment analysis was performed using DAVID server^88,89^. GO terms were identified using the UniProt server.

### Quantification and statistical analysis

The mean values were calculated from the respective cells. Plots of cells are made using MATLAB and boxplots are generated using R. Kruskal–Wallis Test was done following Dunn’s test in R. ns: p > 0.05, *: p <  = 0.05, **: p <  = 0.01, ***: p <  = 0.001, ****: p <  = 0.0001. A total of 25 different cells per condition were measured and seeded onto different samples.

### Structural modelling

Molecular graphics and analyses were performed using UCSF ChimeraX, developed by the Resource for Biocomputing, Visualization, and Informatics at the University of California, San Francisco, with support from the National Institutes of Health R01-GM129325 and the Office of Cyber Infrastructure and Computational Biology, National Institute of Allergy and Infectious Diseases^90^. To generate structural models of the talin-vinculin-actin complex, we started from an elongated talin model obtained by Benjamin Gould, the full-length vinculin structure (pdb code 1tr2;^91^) with added missing loop residues using Modeller^92^ using standard settings, and an electron microscopy-based model of the complex of the vinculin tail and F-actin (pdb-code 3jbi^93^). The orientation of the membrane relative to the talin FERM domain was based on the structure of an integrin b/talin complex (PDB code 3g9w^51^) and membrane prediction from the OPM database. The relative orientation of the vinculin head and talin was based on the experimental structure of the VBS3/talin complex (PDB code 1rkc^94^). We superimposed matching domains using the matchmaker function of ChimeraX and adapted the flexible linkers between the domains using the loop optimization routine implemented in Modeller. We then interactively positioned the respective domains of talin, vinculin, and actin at the measured distance from the membrane plane, and positioned the rod domains manually between the ABS and IBS. The relative orientation of IBS and the membrane is unknown because of the lack of an experimental structure for the IBS2/integrin complex. Therefore, we positioned rod 11 manually close to the membrane plane to analyze the scaling behavior. Afterwards, all loops and, if needed, rod domains were treated as flexible loops in Modeller to obtain the final models.

## Supplementary Information

Supplementary Information.

## Data Availability

The datasets generated in the current study are available from the corresponding author upon reasonable request.

## Abbreviations

ABS: Actin binding site
FA: Focal adhesion
FLIM: Fluorescence lifetime imaging microscopy
H_2_O_2_: Hydrogen peroxide
IBS: Integrin binding site
MIET: Metal-induced energy transfer
ROCK: Rho-associated coiled-coil containing protein kinase
ROS: Reactive oxygen species
VBS: Vinculin binding site

## References

[1] Senoo H, Hata R (1994). Extracellular matrix regulates cell morphology, proliferation, and tissue formation. J. Anat..

[2] Discher DE, Janmey P, Wang Y-L (2005). Tissue cells feel and respond to the stiffness of their substrate. Sci. (N. Y., N.Y.).

[3] Sheetz MP, Felsenfeld DP, Galbraith CG (1998). Cell migration: Regulation of force on extracellular-matrix-integrin complexes. Trends Cell Biol..

[4] Schmitz J, Gottschalk K-E (2008). Mechanical regulation of cell adhesion. Soft Matter.

[5] Zamir, E. & Geiger, B. In *Encyclopedia of Biological Chemistry* (eds. Lennarz, W. J. & Lane, M. D.)128–133 (Elsevier, 2004).

[6] Kanchanawong P (2010). Nanoscale architecture of integrin-based cell adhesions. Nature.

[7] Case LB (2015). Molecular mechanism of vinculin activation and nanoscale spatial organization in focal adhesions. Nat. Cell Biol..

[8] Burridge K, Mangeat P (1984). An interaction between vinculin and talin. Nature.

[9] Hotchin NA, Hall A (1995). The assembly of integrin adhesion complexes requires both extracellular matrix and intracellular rho/rac GTPases. J. Cell Biol..

[10] del-Rio A (2009). Stretching single talin rod molecules activates vinculin binding. Sci. (N. Y., N.Y.).

[11] Chang-Chien C-Y, Chou S-H, Lee H-H (2022). Integrin molecular tension required for focal adhesion maturation and YAP nuclear translocation. Biochem. Biophys. Rep.

[12] Galbraith CG, Yamada KM, Sheetz MP (2002). The relationship between force and focal complex development. J. Cell Biol..

[13] Ridley AJ, Hall A (1992). The small GTP-binding protein rho regulates the assembly of focal adhesions and actin stress fibers in response to growth factors. Cell.

[14] Tilghman RW, Parsons JT (2008). Focal adhesion kinase as a regulator of cell tension in the progression of cancer. Semin. Cancer Biol..

[15] Martino F, Perestrelo AR, Vinarský V, Pagliari S, Forte G (2018). Cellular mechanotransduction: From tension to function. Front. Physiol..

[16] Grandy C, Port F, Pfeil J, Gottschalk K-E (2022). Influence of ROCK pathway manipulation on the actin cytoskeleton height. Cells.

[17] Park JT (2018). A crucial role of ROCK for alleviation of senescence-associated phenotype. Exp. Gerontol..

[18] Kuilman T, Michaloglou C, Mooi WJ, Peeper DS (2010). The essence of senescence. Genes Dev..

[19] Kloc M (2022). Giant multinucleated cells in aging and senescence—an abridgement. Biology.

[20] Jeyapalan JC, Ferreira M, Sedivy JM, Herbig U (2007). Accumulation of senescent cells in mitotic tissue of aging primates. Mech. Ageing Dev..

[21] Pospelova TV, Chitikova ZV, Pospelov VA (2013). Cell Senescence.

[22] Coppé J-P, Desprez P-Y, Krtolica A, Campisi J (2010). The senescence-associated secretory phenotype: The dark side of tumor suppression. Annu. Rev. Pathol..

[23] Kuilman T (2008). Oncogene-induced senescence relayed by an interleukin-dependent inflammatory network. Cell.

[24] Narita M (2003). Rb-mediated heterochromatin formation and silencing of E2F target genes during cellular senescence. Cell.

[25] Hayflick L (1965). The limited in vitro lifetime of human diploid cell strains. Exp. Cell Res..

[26] Hayflick L, Moorhead PS (1961). The serial cultivation of human diploid cell strains. Exp. Cell Res..

[27] Venkataraman K, Khurana S, Tai TC (2013). Oxidative stress in aging–matters of the heart and mind. Int. J. Mol. Sci..

[28] Dimozi A, Mavrogonatou E, Sklirou A, Kletsas D (2015). Oxidative stress inhibits the proliferation, induces premature senescence and promotes a catabolic phenotype in human nucleus pulposus intervertebral disc cells. Eur. Cells Mater..

[29] Duan J, Duan J, Zhang Z, Tong T (2005). Irreversible cellular senescence induced by prolonged exposure to H2O2 involves DNA-damage-and-repair genes and telomere shortening. Int. J. Biochem. Cell Biol..

[30] Facchin F (2018). Comparison of oxidative stress effects on senescence patterning of human adult and perinatal tissue-derived stem cells in short and long-term cultures. Int. J. Med. Sci..

[31] Kiyoshima T (2012). Oxidative stress caused by a low concentration of hydrogen peroxide induces senescence-like changes in mouse gingival fibroblasts. Int. J. Mol. Med..

[32] Lee KS, Lin S, Copland DA, Dick AD, Liu J (2021). Cellular senescence in the aging retina and developments of senotherapies for age-related macular degeneration. J Neuroinflammation.

[33] Martínez-Cué C, Rueda N (2020). Cellular senescence in neurodegenerative diseases. Front. Cell. Neurosci..

[34] Schroth J, Thiemermann C, Henson SM (2020). Senescence and the aging immune system as major drivers of chronic kidney disease. Front. Cell Dev. Biol..

[35] Yang J, Liu M, Hong D, Zeng M, Zhang X (2021). The paradoxical role of cellular senescence in cancer. Front. Cell Dev. Biol..

[36] Liguori I (2018). Oxidative stress, aging, and diseases. Clin. Interv. Aging.

[37] Chizhik AI, Rother J, Gregor I, Janshoff A, Enderlein J (2014). Metal-induced energy transfer for live cell nanoscopy. Nature Photon..

[38] Chizhik AI, Enderlein J (2020). Nanoscale Photonic Imaging.

[39] Lakowicz JR (1999). Principles of Fluorescence Spectroscopy.

[40] Enderlein J (1999). Single-molecule fluorescence near a metal layer. Chem. Phys..

[41] Chizhik AM (2017). Three-dimensional reconstruction of nuclear envelope architecture using dual-color metal-induced energy transfer imaging. ACS Nano.

[42] Chizhik AM (2018). Dual-color metal-induced and Förster resonance energy transfer for cell nanoscopy. Mol. Biol. Cell.

[43] Cho KA (2004). Morphological adjustment of senescent cells by modulating caveolin-1 status. J. Biol. Chem..

[44] Chala N (2021). Mechanical fingerprint of senescence in endothelial cells. Nano Lett..

[45] Kojer K (2012). Glutathione redox potential in the mitochondrial intermembrane space is linked to the cytosol and impacts the Mia40 redox state. EMBO J..

[46] Kovács M, Tóth J, Hetényi C, Málnási-Csizmadia A, Sellers JR (2004). Mechanism of blebbistatin inhibition of myosin II. J. Biol. Chem..

[47] Gnanasambandam R (2017). GsMTx4: Mechanism of Inhibiting mechanosensitive ion channels. Biophys. J..

[48] Liu J (2015). Talin determines the nanoscale architecture of focal adhesions. Proc. Natl. Acad. Sci. U.S.A..

[49] Goult BT (2013). RIAM and vinculin binding to talin are mutually exclusive and regulate adhesion assembly and turnover. J. Biol. Chem..

[50] Lomize MA, Pogozheva ID, Joo H, Mosberg HI, Lomize AL (2012). OPM database and PPM web server: Resources for positioning of proteins in membranes. Nucleic Acids Res..

[51] Anthis NJ (2009). The structure of an integrin/talin complex reveals the basis of inside-out signal transduction. EMBO J..

[52] Moes M (2007). The integrin binding site 2 (IBS2) in the talin rod domain is essential for linking integrin beta subunits to the cytoskeleton. J. Biol. Chem..

[53] Ellis SJ, Pines M, Fairchild MJ, Tanentzapf G (2011). In vivo functional analysis reveals specific roles for the integrin-binding sites of talin. J. Cell Sci..

[54] Atherton P (2015). Vinculin controls talin engagement with the actomyosin machinery. Nat. Commun..

[55] Kumar A (2016). Talin tension sensor reveals novel features of focal adhesion force transmission and mechanosensitivity. J. Cell Biol..

[56] Yao M (2016). The mechanical response of talin. Nat. Commun..

[57] Hytönen VP, Vogel V (2008). How force might activate talin's vinculin binding sites: SMD reveals a structural mechanism. PLoS Comput. Biol..

[58] Gingras AR (2005). Mapping and consensus sequence identification for multiple vinculin binding sites within the talin rod. J. Biol. Chem..

[59] Tapia-Rojo R, Alonso-Caballero A, Fernandez JM (2020). Direct observation of a coil-to-helix contraction triggered by vinculin binding to talin. Sci. Adv..

[60] Barnett SFH, Goult BT (2022). The MeshCODE to scale-visualising synaptic binary information. Front. Cell Neurosci..

[61] Yao M (2014). Mechanical activation of vinculin binding to talin locks talin in an unfolded conformation. Sci. Rep..

[62] Huang DL, Bax NA, Buckley CD, Weis WI, Dunn AR (2017). Vinculin forms a directionally asymmetric catch bond with F-actin. Sci. (N. Y., Ny..

[63] Kluger C (2020). Different vinculin binding sites use the same mechanism to regulate directional force transduction. Biophys. J..

[64] Turner CE, Glenney JR, Burridge K (1990). Paxillin: A new vinculin-binding protein present in focal adhesions. J. Cell Biol..

[65] Yao M (2022). Force- and cell state-dependent recruitment of Piezo1 drives focal adhesion dynamics and calcium entry. Sci. Adv..

[66] Hu X, Margadant FM, Yao M, Sheetz MP (2017). Molecular stretching modulates mechanosensing pathways. Protein Sci..

[67] Illario M (2003). Calcium/calmodulin-dependent protein kinase II binds to Raf-1 and modulates integrin-stimulated ERK activation. J. Biol. Chem..

[68] Blystone SD, Slater SE, Williams MP, Crow MT, Brown EJ (1999). A molecular mechanism of integrin crosstalk: Alphavbeta3 suppression of calcium/calmodulin-dependent protein kinase II regulates alpha5beta1 function. J Cell Biol.

[69] Mizuno Y (2008). Myosin light chain kinase activation and calcium sensitization in smooth muscle in vivo. Am. J. Physiol. Cell Physiol..

[70] Chinthalapudi K, Rangarajan ES, Izard T (2018). The interaction of talin with the cell membrane is essential for integrin activation and focal adhesion formation. Proc. Natl. Acad. Sci. U.S.A..

[71] Braeutigam A, Simsek AN, Gompper G, Sabass B (2022). Generic self-stabilization mechanism for biomolecular adhesions under load. Nat Commun.

[72] Grandy C (2023). Cell shape and tension alter focal adhesion structure. Biomater.Adv..

[73] Ranade SS (2014). Piezo1, a mechanically activated ion channel, is required for vascular development in mice. Proc. Natl. Acad. Sci. U.S.A..

[74] Liu H (2022). Piezo1 channels as force sensors in mechanical force-related chronic inflammation. Front. Immunol..

[75] Jetta D, Bahrani Fard MR, Sachs F, Munechika K, Hua SZ (2021). Adherent cell remodeling on micropatterns is modulated by Piezo1 channels. Sci Rep.

[76] Alge CS, Hauck SM, Priglinger SG, Kampik A, Ueffing M (2006). Differential protein profiling of primary versus immortalized human RPE cells identifies expression patterns associated with cytoskeletal remodeling and cell survival. J. Proteome Res..

[77] Pan C, Kumar C, Bohl S, Klingmueller U, Mann M (2009). Comparative proteomic phenotyping of cell lines and primary cells to assess preservation of cell type-specific functions. Mol. Cell. Proteom..

[78] Shin E-Y (2020). Integrin-mediated adhesions in regulation of cellular senescence. Sci. Adv..

[79] Rapisarda V (2017). Integrin beta 3 regulates cellular senescence by activating the TGF-β pathway. Cell Rep..

[80] Grandy C, Kolb P, Port F, Gottschalk K-E (2020). Micropatterning of cells on gold surfaces for biophysical applications. STAR Protocols.

[81] Ruoslahti E, Pierschbacher MD (1987). New perspectives in cell adhesion: RGD and integrins. Sci. (N. Y., N.Y.).

[82] Gilbert HTJ (2019). Nuclear decoupling is part of a rapid protein-level cellular response to high-intensity mechanical loading. Nat. Commun..

[83] Pavlovic E, Quist AP, Gelius U, Oscarsson S (2002). Surface functionalization of silicon oxide at room temperature and atmospheric pressure. J. Colloid Interface Sci..

[84] Curl CL (2005). Refractive index measurement in viable cells using quantitative phase-amplitude microscopy and confocal microscopy. Cytometry Part A J. Int. Soc. Anal. Cytol..

[85] Radzinski M (2018). Temporal profiling of redox-dependent heterogeneity in single cells. Elife.

[86] Cox J, Mann M (2008). MaxQuant enables high peptide identification rates, individualized p.p.b.-range mass accuracies and proteome-wide protein quantification. Nature Biotechnol..

[87] Tyanova S (2016). The Perseus computational platform for comprehensive analysis of (prote)omics data. Nat. Methods.

[88] Sherman BT (2021). DAVID: A web server for functional enrichment analysis and functional annotation of gene lists (2021 update). Nucleic Acids Res..

[89] Da Huang W, Sherman BT, Lempicki RA (2009). Systematic and integrative analysis of large gene lists using DAVID bioinformatics resources. Nat. Protoc..

[90] Pettersen EF (2021). UCSF ChimeraX: Structure visualization for researchers, educators, and developers. Protein Sci..

[91] Borgon RA, Vonrhein C, Bricogne G, Bois PRJ, Izard T (2004). Crystal structure of human vinculin. Struct. (Lond. Engl.: 1993).

[92] Webb B, Sali A (2016). Comparative protein structure modeling using modeller. Curr. Protocols Bioinform..

[93] Kim LY (2016). The structural basis of actin organization by vinculin and metavinculin. J. Mol. Biol..

[94] Izard T (2004). Vinculin activation by talin through helical bundle conversion. Nature.

