# Supplementary material for: Remodeling of the focal adhesion complex by hydrogen-peroxide-induced senescence
Source: Sci Rep. 2023 Jun 15;13:9735. doi: 10.1038/s41598-023-36347-2 (PMC10272183; doi:10.1038/s41598-023-36347-2)
Supplement: Supplementary file 1 — Supplementary Information. [file 41598_2023_36347_MOESM1_ESM.docx]

Remodeling of the focal adhesion complex by hydrogen-peroxide-induced senescence - Supplementary

Scientific Reports – Special Issue Mechanotransduction

Carolin Grandy^1^, Fabian Port^1^, Meytal Radzinski^3^, Karmveer Singh^2^, Dorothee Erz^1^, Jonas Pfeil^1^, Dana Reichmann^3^, Kay-Eberhard Gottschalk^1,*^

^1^Ulm University, Institute of Experimental Physics, Ulm, Baden-Württemberg, 89081, Germany

^2^Ulm University, **Department of Dermatology and Allergic Diseases, Ulm**, Baden-Württemberg, 89081, Germany

^3^The Hebrew University of Jerusalem, Alexander Silberman Institute of Life Science, Edmond J. Safra Campus - Givat Ram, Jerusalem 9190401, Israel

^*^Correspondence: [kay.gottschalk@uni-ulm.de](mailto:kay.gottschalk@uni-ulm.de)

Table 1S: Height (mean ± std) actin, paxillin, vinculin, talin, membrane of wild type and senescent fibroblasts (Untreated, Blebbistatin, RhoActivator, GsMTx4)

|  | Wild-type | | | | Senescent | | | | |
| --- | --- | --- | --- | --- | --- | --- | --- | --- | --- |
|  | U | B | R | G | | U | B | R | G |
| Actin Height (nm) | 102 ± 8 | 93 ± 8 | 124 ± 10 | 75 ± 9 | | 82 ± 8 | 72 ± 7 | 62 ± 8 | 73 ± 9 |
| Paxillin Height (nm) | 43 ± 3 | 44 ± 3 | 46 ± 3 | 47 ± 3 | | 37 ± 4 | 46 ± 2 | 41 ± 3 | 45 ± 4 |
| Vinculin Height (nm) | 43 ± 2 | 47 ± 3 | 49 ± 3 | 45 ± 3 | | 47 ± 3 | 46 ± 5 | 41 ± 4 | 42 ± 4 |
| Talin Height (nm) | 53 ± 3 | 45 ± 2 | 47 ± 3 | 41 ± 3 | | 36 ± 3 | 49 ± 6 | 39 ± 4 | 44 ± 4 |
| Membrane Height (nm) | 30 ± 5 | 27 ± 5 | 37 ± 6 | 31 ± 4 | | 32 ± 8 | 28 ± 3 | 25 ± 5 | 34 ± 6 |


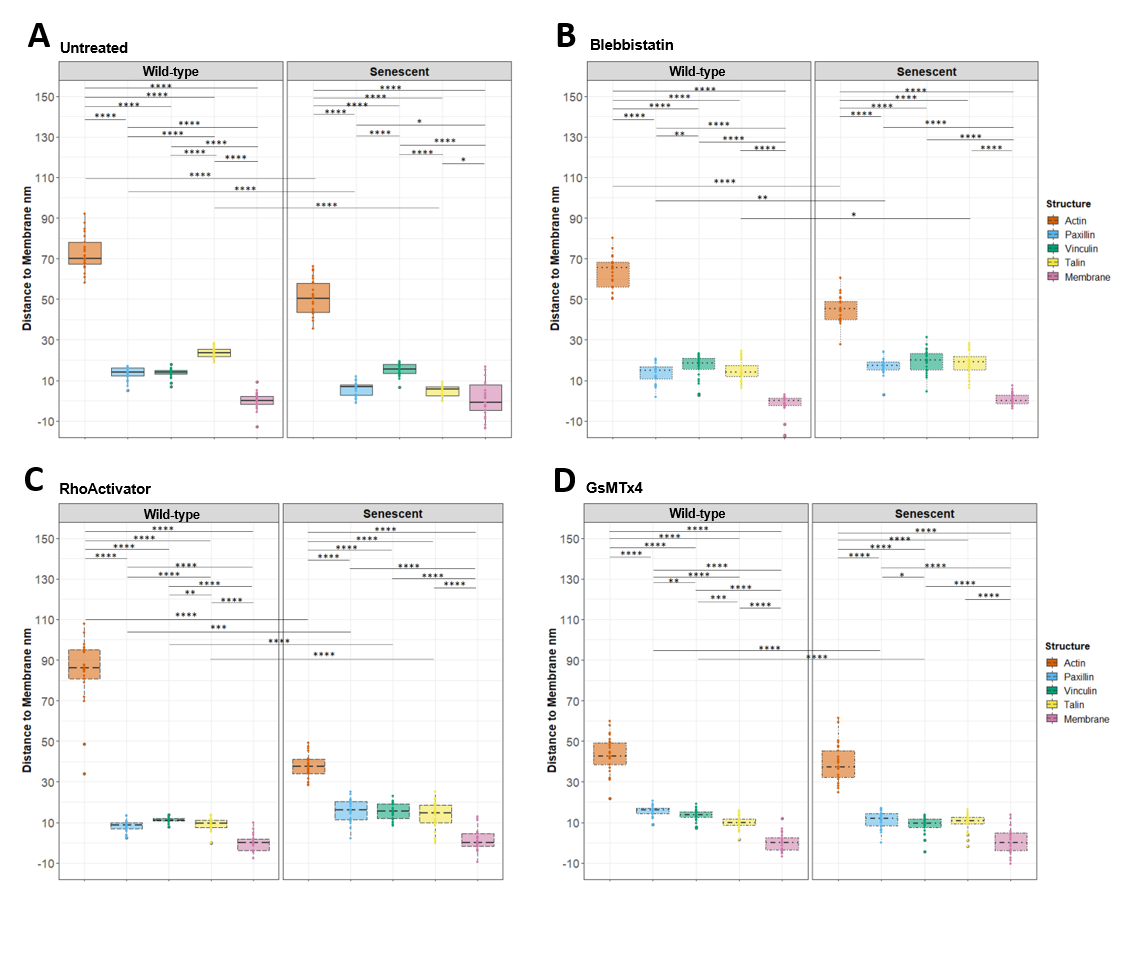


Figure 1S: Distance to the membrane height of wild-type and senescent fibroblasts. A. Untreated, B. Blebbistatin, C. RhoActivator, D. GsMTx4. Statistical test: Kruskal-Wallis Test with post hoc Dunn’s test, p > 0.05, *: p < = 0.05, **: p < = 0.01, ***: p < = 0.001, ****: p < = 0.0001, N=25.


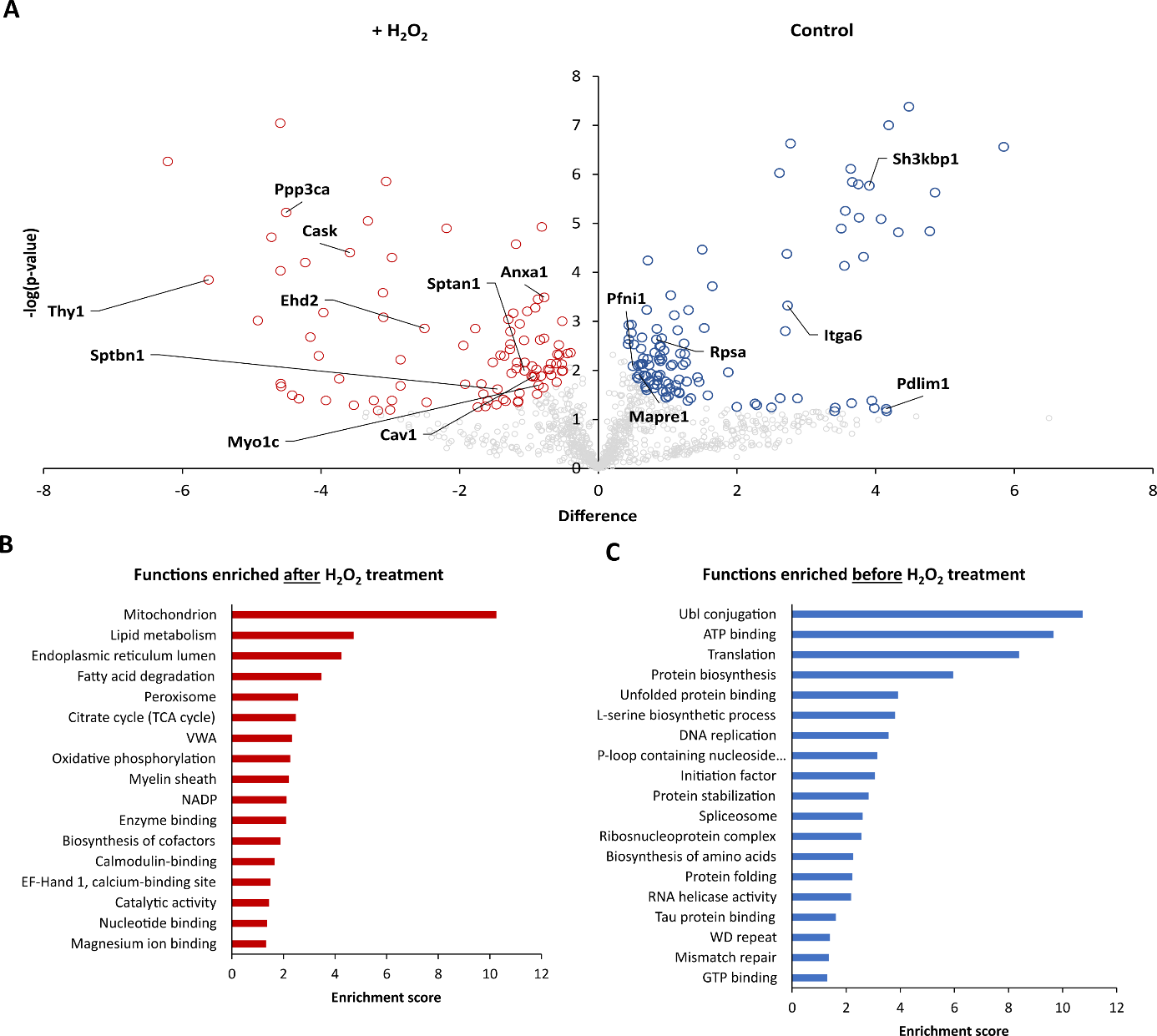


Figure 2S: Analysis of changes in proteomic profiles of wild-type and senescent cells.


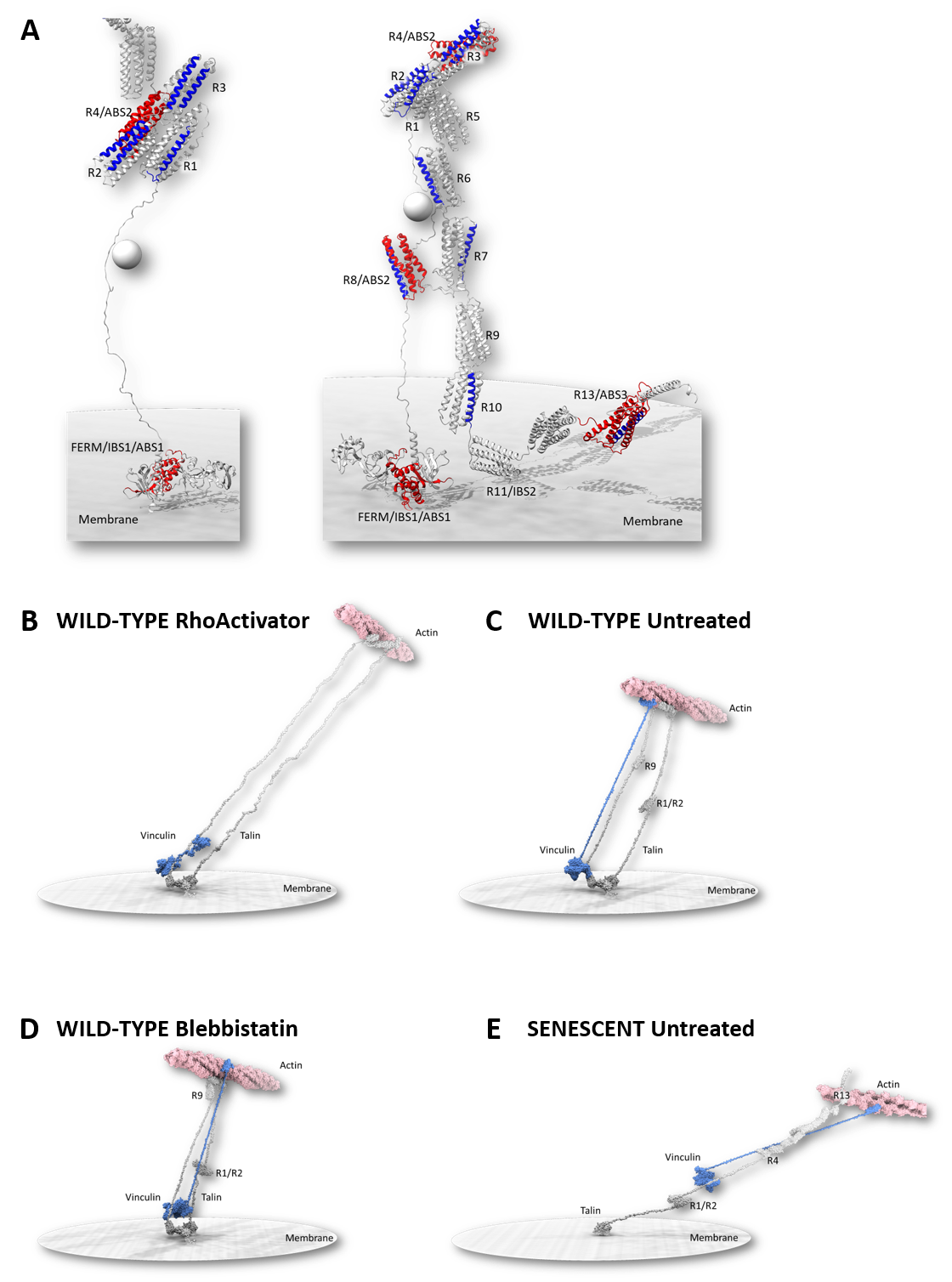


Figure 3S: A) Talin on membrane. Actin binding sites are red, vinculin binding sites are blue, and the centroid position of the approximate epitope for the talin antibody is shown as a sphere. When only IBS1 was engaged, the most membrane-proximal VBS was above the talin epitope, which contradicts our data. However, when both IBS are engaged (right), the distance between both the VBS and the talin epitope to the membrane is in agreement with our model. However, ABS2 needs to be elevated, which requires the unfolding of rod-domain helices. B-E: Conformations in agreement with our data. An increase in strain requires the unfolding of rod domains, whereas a decrease in strain allows refolding of the domains. In senescent cells, the data corroborate a completely different binding mode, with only IBS1 engaged and actin bound to ABS3.
